# Isocitrate dehydrogenase 1 mutations drive downregulation of IL1R1 and dysregulated inflammatory response in acute myeloid leukemia

**DOI:** 10.1038/s41408-025-01445-z

**Published:** 2026-01-06

**Authors:** Sophie Steinhäuser, Thomas Beder, Emely Hübner, Dorothea S. Feuereisel, Jonathan Jebens, Nadine Wolgast, Muhammed Bilal Karaca, Kathrin Richter, Nina Hedemann, Britta Steer, Miriam Denker, Sonja Bendig, Monika Brüggemann, Patricia Silva, Lars Fransecky, Martin Neumann, Friedrich Stölzel, Alina M. Hartmann, Timo Gemoll, Lorenz Bastian, Claudia D. Baldus, Simone Lipinski

**Affiliations:** 1 Department of Internal Medicine II (Hematology/Oncology), University Hospital Schleswig-Holstein, Kiel, Germany; 2 University Cancer Center Schleswig-Holstein (UCCSH), University Hospital Schleswig-Holstein, Kiel and Lübeck, Germany; 3 Hannover Graduate School for Neurosciences, Infection Medicine, and Veterinary Sciences, Hannover, Germany; 4 Clinical Research Unit CATCH ALL (KFO 5010), Kiel, Germany; 5 Department of Gynecology and Obstetrics, Kiel University and University Medical Center Schleswig-Holstein, Kiel, Germany; 6 Charité – Universitätsmedizin Berlin, Corporate Member of Freie Universität Berlin and Humboldt-Universität zu Berlin, and Berlin Institute of Health, Department of Hematology, Oncology, and Cancer Immunology, Berlin, Germany; 7 Section for Translational Surgical Oncology & Biobanking, Department of Surgery, Lübeck University and University Medical Center Schleswig-Holstein, Lübeck, Germany; 8 German Institute of Food Technologies (DIL e.V.), Quakenbrück, Germany; 9 Institute of Food Quality and Food Safety, University of Veterinary Medicine Hannover, Foundation, Hannover, Germany

**Keywords:** Acute myeloid leukaemia, Leukaemia

**To the editor:**

Acute myeloid leukemia (AML) is an aggressive hematologic malignancy characterized by clonal proliferation and impaired differentiation of myeloid progenitor cells [1]. AML predominantly affects older adults and exhibits substantial genetic heterogeneity, including structural chromosomal alterations and recurrent somatic mutations [2]. In the 2022 WHO/ICC classifications, AML entities are defined by recurrent genetic alterations [3, 4]. Among these, mutations in *isocitrate dehydrogenase 1* (*IDH1*) and *IDH2* occur in approximately 10–20% of cases and define a metabolically and epigenetically distinct subgroup [5]. The IDH1 p.R132H mutation produces the oncometabolite 2-hydroxyglutarate, which inhibits α-KG-dependent dioxygenases such as TET enzymes and induces DNA hypermethylation and transcriptional repression, thereby blocking hematopoietic differentiation and promoting leukemogenesis [6]. In addition to cell-intrinsic mutations, AML progression is influenced by extrinsic factors. Among these, the bone marrow niche contributes to a permissive and pro-inflammatory microenvironment that fosters leukemic proliferation and suppresses normal hematopoiesis [7]. A key mediator of this inflammatory milieu is IL-1/IL1R1 signaling, which disrupts hematopoietic stem-cell function and promotes early leukemic expansion [8, 9]. While the metabolic and epigenetic consequences of *IDH1* mutations are well defined, it remains unclear whether *IDH1*-mutant (mut) AML (i) drives distinct intracellular signaling programs and (ii) shows an altered sensitivity to pro-inflammatory conditions in the bone marrow niche, thereby influencing treatment response and disease progression. These findings raise the question of how genetically defined AML subtypes - such as *IDH1*-mut AML - are shaped by inflammatory signals in the bone marrow niche.

To identify genotype-specific signaling pathways of *IDH1-*mut AML, we analyzed gene expression profiles from two independent AML cohorts (BeatAML2.0 and GSE146173; Fig. 1A, S1). Based on differential gene expression analysis (Table S2), gene set enrichment analysis (GSEA) identified pathway-level alterations between *IDH1*-mut and *IDH1*-wt AML samples. Among 39 significantly enriched pathways, 32 (82%) were downregulated in *IDH1*-mut AML, with 22 (69%) related to immune or inflammatory signaling (Fig. S2A). The top three downregulated hallmark pathways - inflammatory response, IL6/JAK/STAT3 signaling and interferon-gamma response - were consistently reduced in both cohorts (Fig. S2B/C). Similarly, in *IL1R1*-low AML cases, a suppression of inflammatory Hallmark and KEGG pathways was observed compared to *IL1R1*-high AML (Fig. S3, Table S3), mirroring the transcriptional profile of *IDH1*-mut AML. Based on the GSEA results from the *IDH1*-mut vs. *IDH1*-wt comparison, integration of the top three hallmark pathways with downregulated genes from the BeatAML2.0 dataset identified 43 overlapping candidates (Fig. S4, Table S4). Protein-protein interaction network analysis (STRING) revealed four functional clusters, including one centered on *IL1R1* and related genes (*IL1R2*, *IL18R1*, *IL18RAP*), highlighting dysregulation of IL1R-family signaling, alongside modules linked to pattern recognition, TNF signaling and interferon responses (Fig. 1B). Comparison across genotypes confirmed that IL1R-family genes were specifically downregulated in *IDH1*-mut AML, but not in *IDH2*-mut cases (Figs. 1C, S5A). In contrast, genes associated with TNF signaling, pattern recognition and interferon-stimulated pathways were reduced in both *IDH1*- and *IDH2*-mut AML (Fig. S5B–D). Given the downregulation and shared chromosomal locus of IL1R-family genes (Fig. S6A), we hypothesized coordinated epigenetic regulation of this specific locus. DNA methylation profiling confirmed significant hypermethylation of the *IL1R* locus in *IDH1*-mut AML, with 20 of 73 CpG sites (27%) differentially methylated (Fig. S6B–E, Table S5). To assess clinical relevance of *IL1R1* expression, AML patients were stratified into *IL1R1* expression tertiles. For patients that received intensive induction therapy (*n* = 402; Fig. S7A) genotype-stratified analysis showed significant higher complete remission (CR) rates in the *IL1R1*-low group for both *IDH1*-wt and *IDH1*-mut AML (Fig. 1D). Patients with low *IL1R1* expression also had significantly longer overall survival (OS) (low: median 1373 vs. medium 475 vs. high 439 days; *p* < 0.001) (Fig. 1E). *IDH1* mutations were significantly enriched in the *IL1R1*-low group (14.9% vs. 7.5% vs. 3%; *p* = 0.002), however *IDH1* mutation status alone did not affect survival (Fig. S7B). In a multivariable Cox model including age and ELN 2022 risk groups, *IL1R1* expression as continuous variable was independently associated with OS (*p* < 0.001; Fig. 1F). The same prognostic association was observed when the analysis was extended to the entire cohort of intensive and non-intensively treated AML patients (n = 583), confirming *IL1R1* as an independent marker of favorable outcome in AML (Fig. S7C/D). Together, these findings link the *IDH1-*mut genotype to IL1R-family gene downregulation and hypermethylation. Low *IL1R1* expression, enriched in *IDH1-*mut AML, was associated with improved treatment response and survival, suggesting that reduced IL1R-mediated signaling is clinically relevant in *IDH1*-mut AML.

**Fig. 1:**
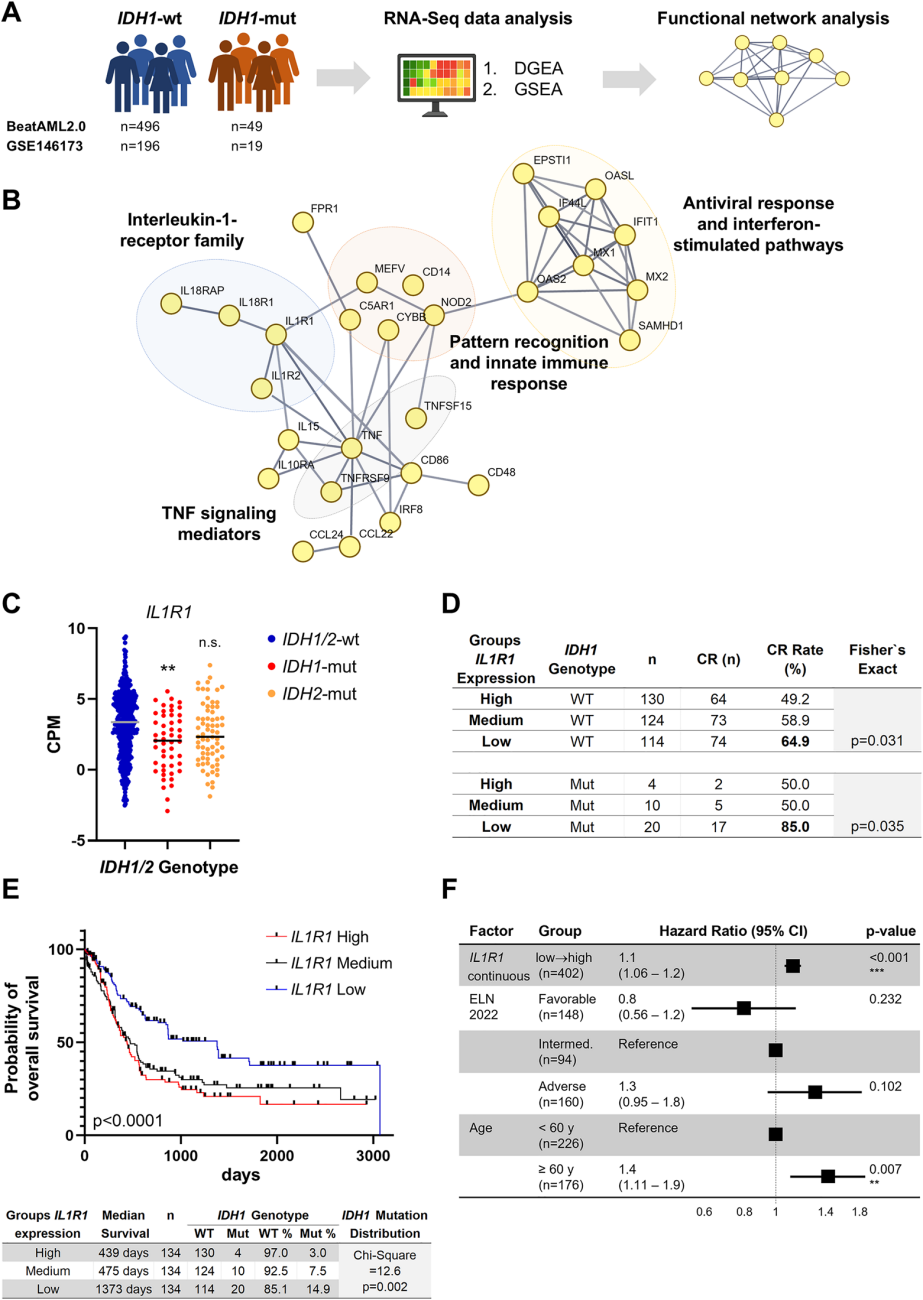
Dysregulated inflammatory signaling and clinical impact of *IL1R1* expression in AML. **A** Overview of cohort composition and RNA-seq-based differential gene expression and pathway analyses. **B** Network analysis of candidate genes from downregulated immune response pathways in *IDH1*-mut vs. *IDH1-*wt AML. Yellow nodes represent individual genes, highlighting functional modules involving IL1R-family members, TNF signaling mediators, pattern recognition and innate immune response, and interferon-stimulated antiviral pathways. **C** *IL1R1* mRNA expression levels (RNA-seq) in *IDH1/2-wt*, *IDH1-mut*, and *IDH2-mut* AML samples from the BeatAML2.0 cohort. Each dot represents one sample (*IDH1/2*-wt, *n* = 497; *IDH1*-mut, *n* = 49; *IDH2*-mut, *n* = 69). p-values from *t*-tests; **p < 0.01; n.s., not significant. **D**–**F** Analyses restricted to intensively treated patients (n = 402) from the BeatAML2.0 cohort. **D** Complete remission (CR) rates across *IL1R1* expression tertiles (low, medium, high) for *IDH1-wt* and *IDH1-mut* AML. For *IDH1*-wt and *IDH1*-mut genotype groups, the number of patients (n), number achieving CR (n), CR rates (%) and corresponding *p*-values from Fisher’s exact test comparing the low expression group versus the combined medium and high groups are shown. **E** Kaplan-Meier analysis of OS by *IL1R1* expression tertiles. P-value was calculated using the log-rank test. Tables below indicate median survival, number of patients (n) and *IDH1* mutation distribution in each expression group. Differences in *IDH1* mutation frequency across expression groups were assessed using the chi-square test. **F** Multivariable Cox regression for OS including *IL1R1* expression (continuous), age (≥60 vs. <60 y) and ELN 2022 risk classification. Hazard ratios with 95% confidence intervals are shown. For this model, the number of events was 237; the global *p*-value (log-rank) was 4.3515e−08; AIC was 2495.27; and the concordance index was 0.62.

To explore the functional consequences of reduced *IL1R1* expression in *IDH1*-mut AML, primary blasts (Table S6) were stimulated with IL-1β. Under basal conditions, *IDH1*-mut AML blasts showed reduced *IL1R1* expression (Fig. S8, Table S7). Upon IL-1β stimulation, *IDH1*-mut blasts displayed markedly reduced induction of inflammatory mediators, including *TNF*, *IL6*, and *CCL20*, compared with *IDH1*-wt (Fig. S9A–D, Table S7), consistent with impaired NF-κB pathway activation. GSEA confirmed downregulation of TNF-α signaling via NF-κB and inflammatory response pathways (Fig. S10). For functional validation, we used the modified KG-1a cell line heterozygous for the *IDH1* R132H mutation (*IDH1*-het) [10]. Consistent with patient data, *IL1R1* expression was significantly reduced in *IDH1*-het compared to *IDH1*-wt cells, both under basal conditions and after IL-1β stimulation (Fig. S11A). Inflammatory protein profiling using Olink® showed significantly lower secretion of IL-1β-induced NF-κB target chemokines in *IDH1*-het cells (Fig. 2A), but selective upregulation of TRAIL (TNFSF10) and other immune modulators. Protein validation by ELISA confirmed distinct secretion patterns: IL-8 was strongly induced in *IDH1*-wt but not in *IDH1*-het cells, PLAU in both and TRAIL selectively in *IDH1*-het cells (Fig. S11B/C). Given its pro-apoptotic role, we tested whether IL-1β induces apoptotic programs in *IDH1*-het cells. GSEA revealed significant enrichment of apoptosis-related signatures in IL-1β-stimulated *IDH1*-het compared to *IDH1*-wt KG-1a cells (Fig. 2B), indicating that *IDH1-mut* reprograms IL-1β-responsive signaling towards a pro-apoptotic phenotype. Cross-cohort transcriptomic analyses revealed reduced basal *TRAIL* mRNA expression in *IDH1*-mut compared with *IDH1*-wt AML in BeatAML2.0 and a similar but nonsignificant trend in GSE146173 (Fig. S12A/B). These findings indicate that TRAIL expression is not constitutively elevated in *IDH1*-mut AML but becomes selectively induced under inflammatory conditions, consistent with the IL-1β-dependent response observed in our experimental models. To further test the functional relevance of IL1R1-dependent TRAIL induction under inflammatory conditions, *IDH1*-wt and *IDH1*-het KG-1a cells were exposed to pro-inflammatory stromal conditioned medium (HS-5 CM). HS-5 CM significantly increased TRAIL secretion and apoptosis in *IDH1*-het cells, both of which were abrogated by the IL1R1 antagonist Anakinra (Figs. 2C, D, S13A). Time-resolved caspase-3/7 activation confirmed accelerated apoptotic kinetics in *IDH1*-het compared with *IDH1*-wt cells under inflammatory conditions (Figs. 2E/F, S13B/C). In primary AML blasts, HS-5 CM induced higher TRAIL and lower TNF levels in *IDH1*-mut samples (Fig. S14A/B) and led to increased caspase-3/7 activation, which was reversed by IL1R1 blockade (Fig. S14C/D). Together, these results demonstrate that *IDH1* mutations are associated with dysregulated IL1R1 signaling, characterized by reduced receptor expression, impaired downstream responses and increased susceptibility to stromal inflammatory signals. This identifies a genotype-linked vulnerability of *IDH1*-mut AML to inflammation-induced cell death through IL1R1 signaling.

**Fig. 2:**
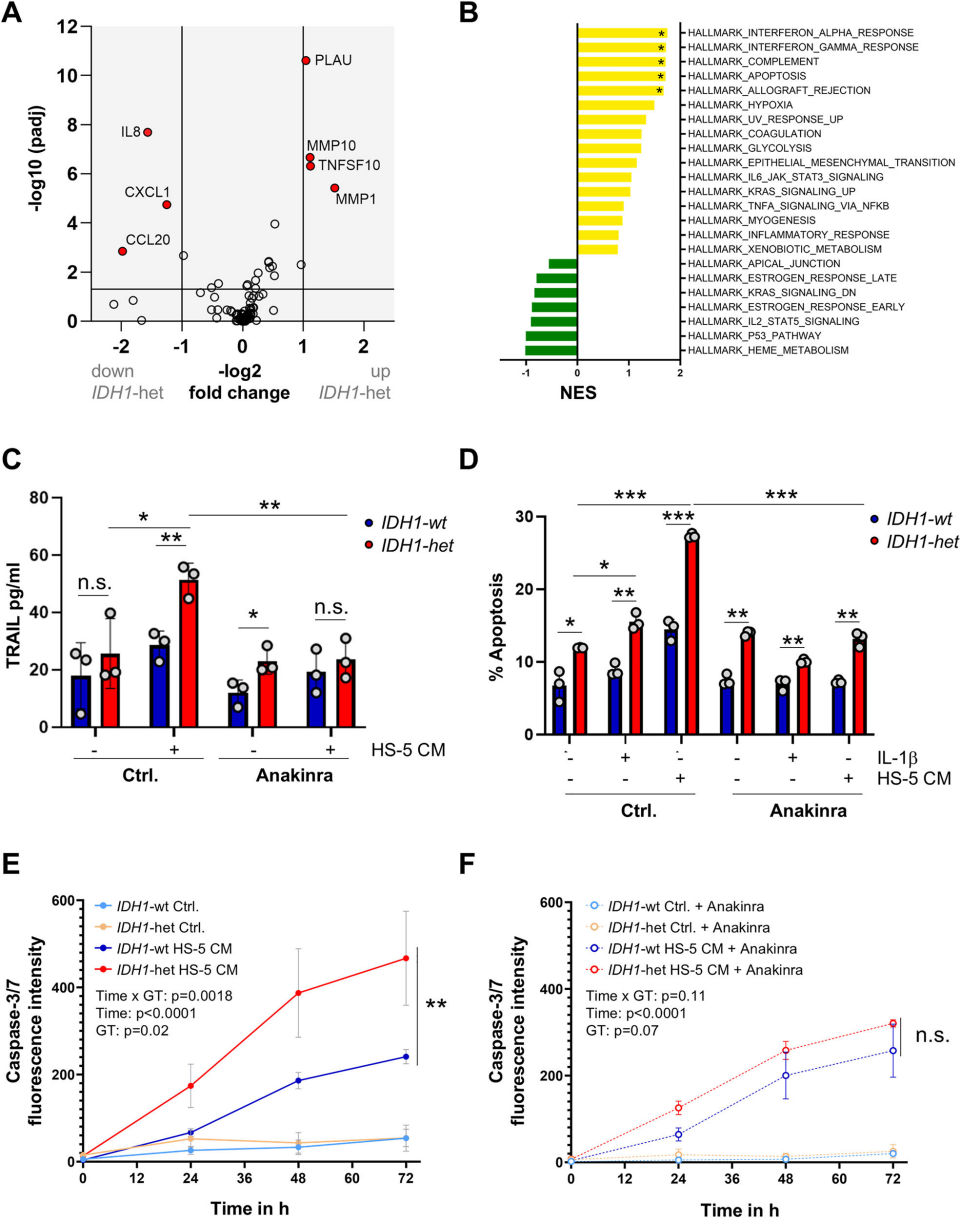
Altered IL1R1 signaling and apoptosis in *IDH1*-mut AML cells. **A** Volcano plot depicting significantly differentially secreted proteins in *IDH1*-het vs. *IDH1*-wt KG-1a cells following IL-1β stimulation (10 ng/mL, 18 h), based on *n* = 6 biological replicates and measured using the Olink® Target 96 Inflammation panel. Dashed horizontal line indicates a -log10 adjusted p-value threshold of 1.3 (*p* = 0.05); vertical dashed lines represent log2 fold change cutoffs of ±1. **B** Bar plot showing GSEA results based on transcriptomic profiles of IL-1β–stimulated *IDH1*-wt and *IDH1*-het KG-1a cells. Selected hallmark gene sets are ranked by NES. Yellow bars indicate positive enrichment, green bars negative enrichment. Asterisks denote significance based on FDR q-values: *q < 0.05. **C** ELISA measurement of TRAIL levels in cell supernatants of *IDH1*-wt and *IDH1*-het KG-1a cells following stimulation with HS-5 CM for 48 h, with or without Anakinra (10 µg/ml) treatment. Data represent mean ± SD from three independent biological replicates. Statistical analysis: unpaired two-tailed t-tests with Šidák correction for multiple comparisons; *p < 0.05, **p < 0.01; n.s. = not significant. **D** Flow cytometric quantification of apoptosis (% Annexin V+ cells) in *IDH1*-wt and *IDH1*-het cells after 48 h of stimulation with 10 ng/ml IL-1β or HS-5 CM, with or without Anakinra (10 µg/ml) treatment. Data represent mean ± SD from three independent biological replicates. Statistical analysis: unpaired two-tailed t-tests with Šidák correction for multiple comparisons; *p < 0.05, **p < 0.01, ***p < 0.001; n.s. = not significant. **E**, **F** Caspase-3/7 activation in *IDH1*-wt and *IDH1*-het KG-1a cells under control conditions or with HS-5 stromal cell–derived conditioned media (CM) over 72 h, assessed by live-cell fluorescence imaging without (**E**) or with 10 µg/ml Anakinra (**F**). Fluorescence was measured at 0, 24, 48 and 72 h. Data represent mean ± SD of *n* = 3 biological replicates. Statistical analysis was performed using two-way repeated measures ANOVA; Geisser-Greenhouse correction was applied in (**E**) due to violation of sphericity. **p < 0.01, n.s. = not significant.

Inflammatory signaling is increasingly recognized as a determinant of AML biology, contributing to disease progression and hematopoietic dysfunction [11], yet, it remains unclear how genetic drivers modulate these pathways. Here we show that *IDH1*-mut AML is characterized by downregulation of inflammatory signaling pathways, consistent with prior reports linking *IDH1* mutations to immune suppression [12]. Low expression levels of *IL1R1*, a central regulator of inflammatory pathways, correlated with improved chemotherapy response and survival in both *IDH1*-wt and *IDH1*-mut AML, with significant enrichment of *IDH1*-mut samples among *IL1R1*-low cases. This extends previous studies identifying high *IL1R1* expression as a negative prognostic marker [13, 14] by showing that *IL1R1*-low status is significantly enriched in *IDH1*-mut AML. Functionally, *IDH1*-mut AML cells showed an apoptosis-prone response to IL-1β or stromal-derived inflammatory stimuli, contrasting the general pro-survival effects of IL-1β in AML. Reduced *IL1R1* expression underpinned this *IDH1*-specific phenotype, as IL1R1 blockade with Anakinra reversed apoptosis in *IDH1*-mut but not *IDH1*-wt cells. At the molecular level, *IDH1*-mut AML showed impaired IL1R1-mediated signaling, reduced NF-κB activation, selective TRAIL induction, and enrichment of pro-apoptotic transcriptional programs. Although additional 2-HG-dependent epigenetic alterations may contribute to this phenotype, the consistent association of low *IL1R1* expression with improved response and survival indicates a biologically relevant vulnerability in *IDH1*-mut AML. Importantly, this does not imply that IL1R1 should be therapeutically inhibited in this subgroup. On the contrary, our data suggest that intact IL1R1 signaling facilitates apoptosis induction in *IDH1*-mut cells under inflammatory conditions, indicating that its activity may in fact contribute to therapeutic efficacy. Accordingly, IL1R antagonists such as Anakinra or canakinumab, currently evaluated in hematologic malignancies (NCT04239157) [7, 15], may require genotype-specific consideration. These findings may also have implications for *IDH1*-targeted therapies such as Ivosidenib, which lower 2-HG levels and promote differentiation.

In conclusion, our data indicate that *IDH1* mutations remodel IL1R-mediated signaling toward an inflammatory-anergic but pro-apoptotic state, revealing a previously unrecognized vulnerability of *IDH1*-mut AML to microenvironmental inflammatory stress. Reduced IL1R signaling may not only predict improved chemotherapy response but also enhance responsiveness to differentiation therapies. Exploiting this vulnerability may guide the development of more effective, genotype-adapted treatment strategies to improve outcomes in *IDH1*-mut AML.

## Supplementary information

Supplementary Material

Supplementary Table 2

Supplementary Table 3

Supplementary Table 7

## Data Availability

The datasets generated and/or analyzed during the current study will be made available from the corresponding author upon reasonable request.
