# Supplementary material for: Isocitrate dehydrogenase 1 mutations drive downregulation of IL1R1 and dysregulated inflammatory response in acute myeloid leukemia
Source: Blood Cancer J. 2026 Jan 6;16(1):5. doi: 10.1038/s41408-025-01445-z (PMC12775398; doi:10.1038/s41408-025-01445-z)
Supplement: Supplementary file 1 — Supplementary Material [file 41408_2025_1445_MOESM1_ESM.docx]

Supplementary Material

*Isocitrate dehydrogenase 1 mutations drive downregulation of IL1R1 and dysregulated inflammatory response in acute myeloid leukemia*

**Supplementary Materials and Methods**

*Cell Culture*

Heterozygous *IDH1* R132H mutant (mut) and *IDH1* wild-type (wt) KG-1a cell clones were previously generated via CRISPR-mediated base editing, as described by Steinhäuser et al. (1) and cultured in RPMI-1640 medium (Gibco, Billings, MT, USA) supplemented with 20% fetal bovine serum (FBS). HS-5 human bone marrow stromal cells were obtained from the American Type Culture Collection (ATCC, Manassas, VA, USA) and cultured in Dulbecco’s Modified Eagle Medium (DMEM; Gibco, Billings, MT, USA) supplemented with 10% FBS and 1% penicillin-streptomycin. HS-5 conditioned medium (CM) was generated by culturing HS-5 cells to 80% confluency, followed by a 24-hour incubation in antibiotic-free medium. The supernatant was subsequently sterile filtered through 0.2 µm syringe filters.

*Patients and primary AML samples*

Mononuclear cells (MNCs) from primary bone marrow samples of AML patients were isolated using the FICOLL density gradient method according to the manufacturer´s protocol and subjected to long-term storage in liquid nitrogen. All patients gave written informed consent for the storage and use of residual material for medical research purposes. The Ethics Committee of the Medical Faculty at Christian-Albrechts-University of Kiel has raised no ethical or legal objections regarding the implementation of the study (reference number D-558/25).

*Reagents and ELISA*

Recombinant human IL-1β (Cat. #11340012) was purchased from ImmunoTools (Friesoythe, Germany). The CellEvent™ Caspase-3/7 Green Detection Reagent (Cat. #C10723) was purchased from Invitrogen™ (Thermo Fisher Scientific, Waltham, MA, USA). The IL-1 receptor antagonist Anakinra (Kineret®, 100 mg/670 µL) was obtained from Swedish Orphan Biovitrum GmbH (Order no.: 40108589). Cytokines (IL-8/CXCL8, TRAIL/TNFSF10, uPA/PLAU) were measured in culture supernatants using DuoSet ELISA Kits from R&D Systems (Minneapolis, MN, USA), including IL-8/CXCL8 (Cat. # DY208), TRAIL/TNFSF10 (Cat. # DY375), u-Plasminogen Activator/Urokinase (PLAU; Cat. # DY1310).

*RNA, cDNA, RT-qPCR*

Total RNA was isolated using the RNeasy Plus Mini Kit (Qiagen, Venlo, Netherlands) according to the manufacturer’s instructions. Reverse transcription was performed using the iScript cDNA Synthesis Kit (Bio-Rad, Hercules, CA, USA) Quantitative real-time PCR (RT-qPCR) was carried with the FastStart Essential DNA Green Master Mix (Roche, Basel, Switzerland) on a LightCycler 96 system (Roche). Primer pairs are listed in Supplementary Table S1. Relative gene expression levels were calculated using the 2^-ΔΔCt method, as described by Schmittgen and Livak(2), according to the MIQE guidelines(3).

*RNA sequencing of primary AML blasts and KG-1a clones*

For whole transcriptomic RNA sequencing, *IDH1*-wt (n=3) and *IDH1*-het (n=3) KG-1a cell clones as well as primary bone marrow samples from patients with *IDH1*-wt (n=3) and *IDH1*-mut (n=3) AML were stimulated with 10 ng/ml IL1-β for 6 h or maintained under unstimulated conditions. RNA was isolated using RNeasy Micro Kit (#74004, Qiagen, Venlo, Netherlands) according to the manufacturer´s protocol. RNA-Seq library preparation was performed using Illumina RNA UD Indexes Set A. Paired-end sequencing was performed on a NextSeq1000 using a 200 cycle P2 flow cell for a coverage 30 million reads per sample. Paired-end RNA-seq data were pre-processed and aligned to the human reference genome hg38.

*Secretome Profiling using Olink®*

Protein Profiling of cell culture supernatants was performed using the Olink® Immuno-Oncology 96-plex panel (Olink Proteomics, Sweden), based on proximity extension assay (PEA) technology. This method enables the detection of low-abundance proteins with high specificity and sensitivity (limit of quantification < 0.1 pg/mL). In brief, 1 µL of supernatant was diluted (1:100) and incubated with antibody pairs conjugated to complementary oligonucleotides. Samples were randomized across assay wells according to the standard Olink sample setup procedure to minimize positional and batch effects. Upon binding to the same target, DNA strands were extended and amplified by qPCR (Primus 96 plus, MWG Biotech). The PCR products were transferred to a 96 Dynamic Array IFC for Gene Expression (Fluidigm, South San Francisco, CA, USA) and combined with assay-specific primers using the IFC control Juno™ (Fluidigm). The amplified DNA reporter sequence for each detected protein was then quantified via high-throughput real-time qPCR on the Fluidigm Biomark™ HD System (Fluidigm)(4, 5). NPX values (log₂ scale) were calculated using Olink® software via a standardized three-step process including ΔCt normalization and assay-specific correction. Values reflect relative protein abundance and were used as provided without further modification.

*Annexin V-FITC/PI Staining and Flow Cytometry*

Apoptosis was assessed using the Annexin V-FITC Kit (Miltenyi Biotec, Bergisch Gladbach, Germany, Cat. No. 130-092-052) according to the manufacturer’s instructions. Samples were analyzed using the MACSQuant Analyzer 10 (Miltenyi Biotec, Bergisch Gladbach, Germany).

*Caspase-3/7 activation using brightfield and fluorescence imaging*

*IDH1*-wt and *IDH1*-het KG-1a cells or primary AML blasts from *IDH1*-wt and *IDH1*-mut patients were stained with CellEvent™ Caspase-3/7 Green detection reagent (Thermo Fisher Scientific, Waltham, MA, USA). Caspase-3/7 activation was measured at 0, 24, 48, and 72 hours (cell lines) or at 0, 12, 24, 36, and 48 hours (primary patient samples) using brightfield and fluorescence imaging with a CELLAVISTA® 4K cell imager (SYNENTEC GmbH, Elmshorn, Germany). The Suspension Cell Count (1F) image analysis application of YT-SOFTWARE^®^ was used to quantify the Caspase-3/7 fluorescence intensity or percentage of caspase 3/7-positive cells (SYNENTEC, Short Note SN-B121-XX-05). At 48 h, propidium iodide (PI) was added to identify late apoptotic or necrotic cells. Classification of apoptotic and necrotic cell populations was performed using the Suspension Cell Count (2F) application of YT-SOFTWARE^®^ (SYNENTEC, Short Note SN-B240-XX-05). The following imaging settings were used: 10x objective; Brightfield: Exciter: Brightfield, Emission filter: Green (530/43 nm); Caspase-3/7: Exciter: Blue (475/28 nm), Emission filter: Green (530/43 nm); PI: Exciter: Lime (562/40 nm), Emission filter: Lime (593/LP nm).

*Protein Interaction Network Analysis*

To explore the functional relationships among candidate genes identified through pathway analysis, a protein-protein interaction (PPI) network was constructed using the STRING database(6) (version 12.0). The analysis included interactions derived from text mining, experiments, curated databases, co-expression, neighborhood, gene fusion and co-occurrence sources. Only interactions with a minimum required confidence score of 0.700 (high confidence) were considered. The network type was set to display full STRING interactions, including both functional and physical associations. To identify functional clusters within the network, Markov clustering (MCL) was performed with an inflation parameter of 4. Of the 43 input genes, 27 were found to be interconnected in the network and formed distinct functional modules. These modules were further refined and interpreted through manual curation based on shared molecular functions and gene co-expression patterns.

*Differential gene expression analysis*

To identify genotype-specific signaling pathways linked to *IDH1-*mut AML, differential gene expression was analyzed in two independent datasets, the BeatAML2.0 RNA-Seq dataset with n=615 AML samples(7) including n=49 AML samples with IDH1-mutations and the GSE146173 dataset(8) including n=19 samples with *IDH1* mutations. Analysis was performed in R 4.3.0 using the package limma (version 3.60.4). Low expressed genes were excluded before the analysis. Differentially expressed genes were considered if FDR ≤ 0.01 and absolute log2-fold change > 1.

Differential gene expression analysis on in-house generated RNAseq data was performed in R 4.3.0 using the DESeq2 package (version 1.40.2). The analysis included comparisons between *IDH1*-mut and *IDH1*-wt primary AML blasts, as well as between *IDH1*-het and *IDH1*-wt KG1a cells. After filtering of low expressed genes, differential expression analysis of protein-coding genes was performed using a model based on the grouping variable (treatment or mutation status). Samples were extracted based on predefined comparison groups including different combinations of cell types, mutation status (IDH1) and treatment conditions (IL-1β). For statistical analysis, a DESeq2 dataset was created using the filtered count data. The comparison groups were defined as a factor variable (Group), either based on treatment condition (Treatment) or mutation status (IDH1). The DESeq model was then applied to identify differentially expressed genes with a contrast analysis performed to compare conditions. Additionally, a manual calculation of log2 fold change (log2FC) was conducted to validate the results. Identified differentially expressed genes were filtered based on statistical significance (adjusted p-value ≤ 0.05 and |log2FC| > 1).

*Gene set enrichment analyses*

To evaluate pathway activity associated with *IDH1* genotype, Gene Set Enrichment Analysis (GSEA) was performed on differentially expressed genes identified between *IDH1*-wt and *IDH1*-mut samples in BeatAML2.0 and GSE146173 as well as in-house RNA-Seq data. Analysis of preranked gene lists was conducted in R 4.3.0 using fgsea (version 1.30.0) or by the GSEA desktop application (version 4.3.3; Broad Institute, Cambridge, MA, USA). Analyses were conducted independently for two experimental settings: gene expression data from primary AML bone marrow blast samples of *IDH1*-mut and *IDH1*-wt patients and from *IDH1*-mut and *IDH1*-wt KG1a cells, treated or untreated with IL-1β. Significance was determined using normalized enrichment scores (NES), nominal p-values and false discovery rate (FDR) q-values, with an FDR < 0.25 considered significant. Results were visualized using the GSEA report module and Enrichment Map tools.

*DNA Methylation*

Genome-wide DNA methylation data from patients with *IDH1*-mut and *IDH1*-wt AML were obtained from previously published datasets. Specifically, methylation profiles from the Study Alliance Leukemia (SAL) elderly AML cohort (*n* = 79) and the TCGA AML cohort (*n* = 194) were accessed for integrative re-analysis, as described by(Silva et al. 2017). Methylation profiling in both cohorts was originally performed using the Infinium® HumanMethylation450 BeadChip platform (Illumina, San Diego, CA, USA), and data are publicly available via the NCBI Gene Expression Omnibus (GSE86409). Methylation β-values were transformed to M-values (logit2 scale) for further analysis. Differential methylation analysis focused on CpG sites within the *IL1R* family locus on chromosome 2 (chr2:101,991,960–102,452,565, hg38), which includes *IL1R2*, *IL1R1*, *IL1RL2*, *IL1RL1*, *IL18R1* and *IL18RAP*. Comparisons between *IDH1*-mutant *IDH1-*wt AML samples were performed using the Mann–Whitney U-test (Wilcoxon rank-sum test) and p-values were adjusted for multiple comparisons using the Benjamini–Hochberg false discovery rate (FDR) correction.

*Survival analysis*

To assess the clinical relevance of IL1R family gene expression, overall survival was analyzed in patient subgroups stratified by tertiles of gene expression (high, medium, low). Expression data and clinical annotations were obtained from the BeatAML2.0 RNA-Seq dataset(7). Pediatric cases were excluded; *IDH2*-mut samples were retained (n = 585). Kaplan–Meier survival curves were generated using GraphPad Prism (version 9.5.1; GraphPad Software, San Diego, CA, USA) and differences in survival between groups were assessed using the log-rank (Mantel–Cox) test(9). Categorical variables, including *IDH1* genotype distribution across expression groups, were analyzed using chi-square tests. A p-value < 0.05 was considered statistically significant.

*Statistical analysis*

Statistical analyses and data visualization were performed using GraphPad Prism (version 9.5.1; GraphPad Software, San Diego, CA, USA), Microsoft Excel 2016 and R version 4.3.0 (R Core Team, 2013). Data were assessed for normality using the Shapiro–Wilk test. For normally distributed data, two-tailed unpaired Student’s t-tests were used to compare two groups and one- or two-way ANOVA followed by Bonferroni post hoc tests were applied for multi-group or time-course comparisons. For non-normally distributed data, the nonparametric Mann–Whitney *U* test or Wilcoxon signed-rank test was used as appropriate. Fisher’s exact test was used to compare CR rates across *IL1R1* and *IL1R2* expression tertiles. Two-sided tests were applied unless stated otherwise; for comparisons within the *IDH1*-mut subgroup, one-sided Fisher’s exact tests were used based on prior hypotheses of increased CR rates in the low expression group. Chi-square tests were applied to assess the distribution of *IDH1* mutation status (mut vs. wt) across the same expression groups and to evaluate associations between *IL1R* and *IL1R2* expression levels and overall survival categories. Multivariate survival analysis was conducted by building a Cox proportional hazard ratio model using the coxph() function from the survival package (version 3.8-3) and visualized using the ggforest() function from the survminer package (version 0.5.0). A *p*-value < 0.05 was considered statistically significant (*p* < 0.05; **p* < 0.01; ***p* < 0.001). All experiments were performed at least in triplicate and error bars represent standard deviation unless stated otherwise.

**Supplementary Figure 1**: Graphical abstract


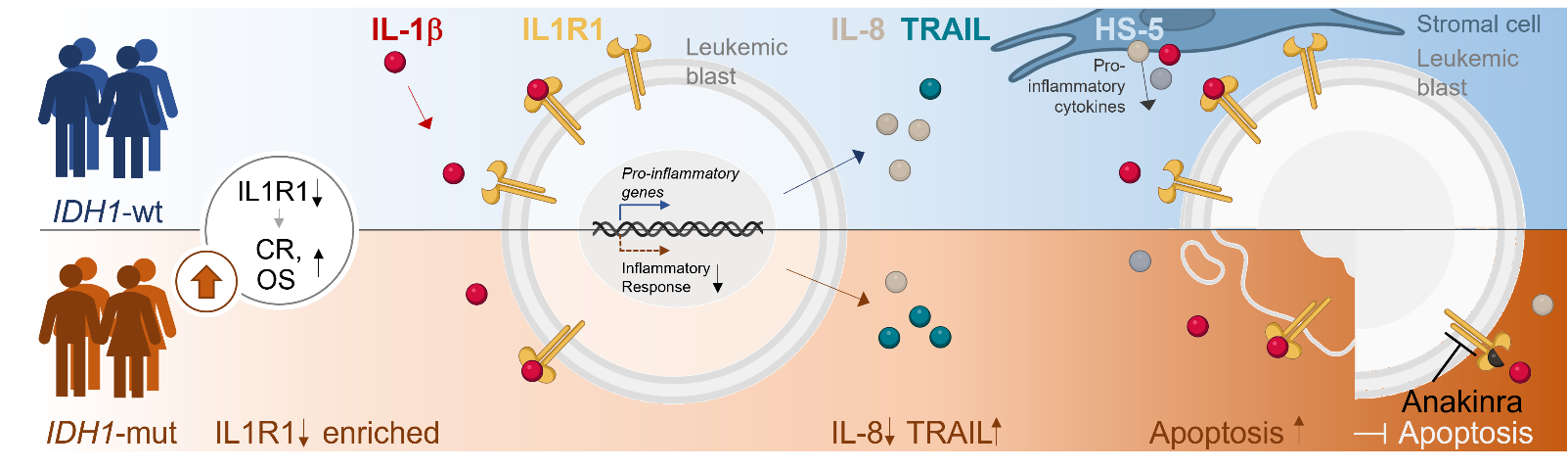


Supplementary Figure1: *IDH1* mutations modulate IL-1β–induced inflammation and sensitize AML blasts to apoptosis. Low *IL1R1* expression is associated with higher complete remission (CR) rates and longer overall survival (OS), with *IDH1*-mut cases showing an enrichment of *IL1R1*-low expression compared to *IDH1*-wt. In *IDH1*-wt cells, IL-1β binding to IL1R1 activates pro-inflammatory gene expression and IL-8 secretion. *IDH1*-mut cells exhibit reduced IL1R1 expression, diminished IL-8 release, but increased TRAIL production, thereby promoting apoptosis. Stromal priming further sensitizes *IDH1*-mut cells, an effect reversed by IL1R1 blockade with Anakinra.

**Supplementary Figure 2**: Identification of downregulated inflammatory signaling pathways in *IDH1*-mut AML


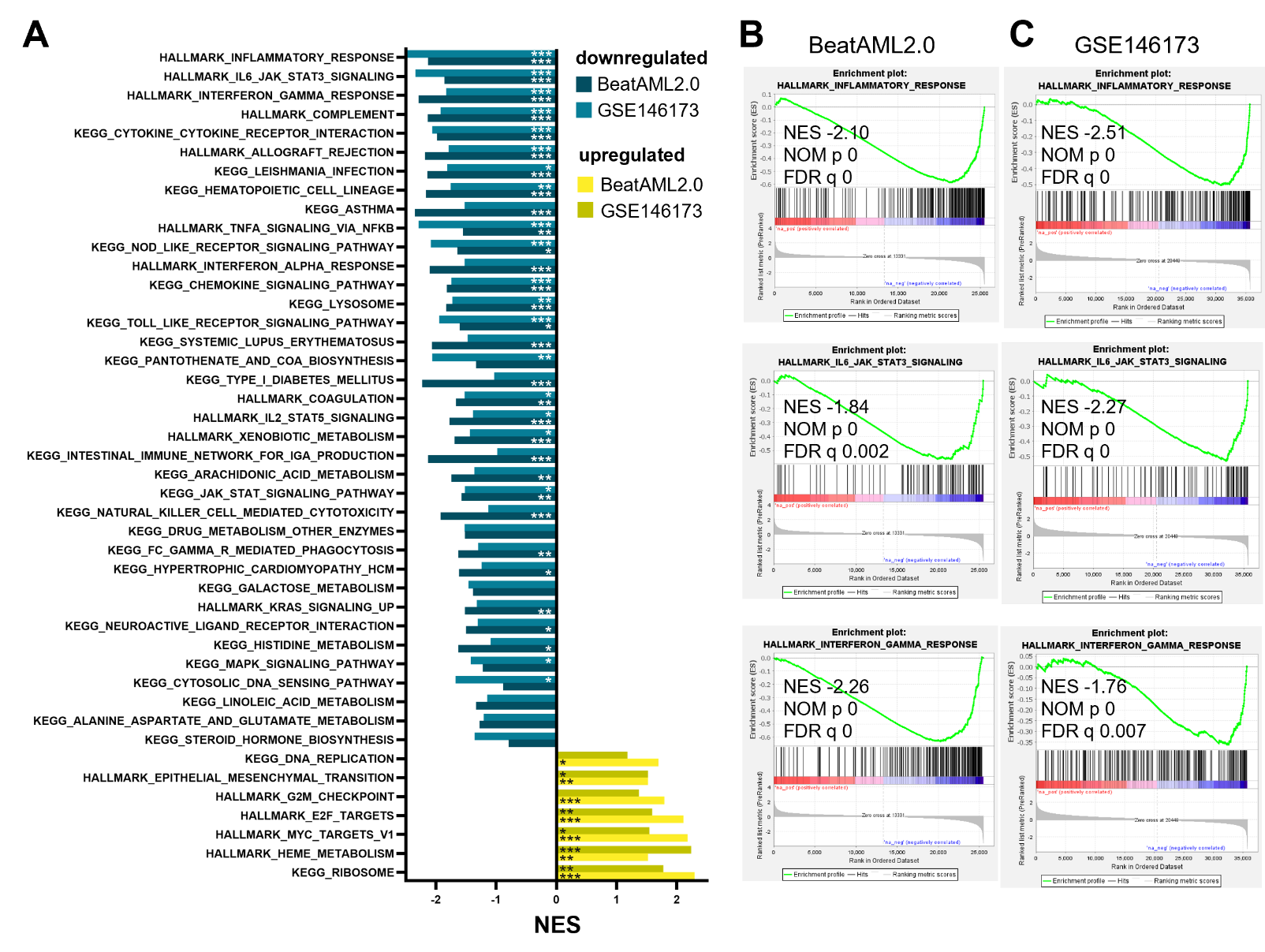


Supplementary Figure 2: (A) Bar plot of Hallmark and KEGG pathway enrichment ranked by normalized enrichment score (NES). Blue bars indicate pathways downregulated in *IDH1*-mut vs. *IDH1*-wt AML; yellow bars indicate upregulated pathways (BeatAML2.0: dark; GSE146173: light). ***p < 0.001, **p < 0.01, *p < 0.05. (B, C) Enrichment plots for the top three negatively enriched Hallmark pathways from the BeatAML2.0 (B) and GSE146173 (C) cohort: inflammatory response (top), IL6/JAK/STAT3 signaling (middle) and interferon gamma response (down). Each plot shows the NES, NOM p, and FDR q-values.

**Supplementary Figure 3**: Pathway enrichment linked to *IL1R1* downregulation in AML


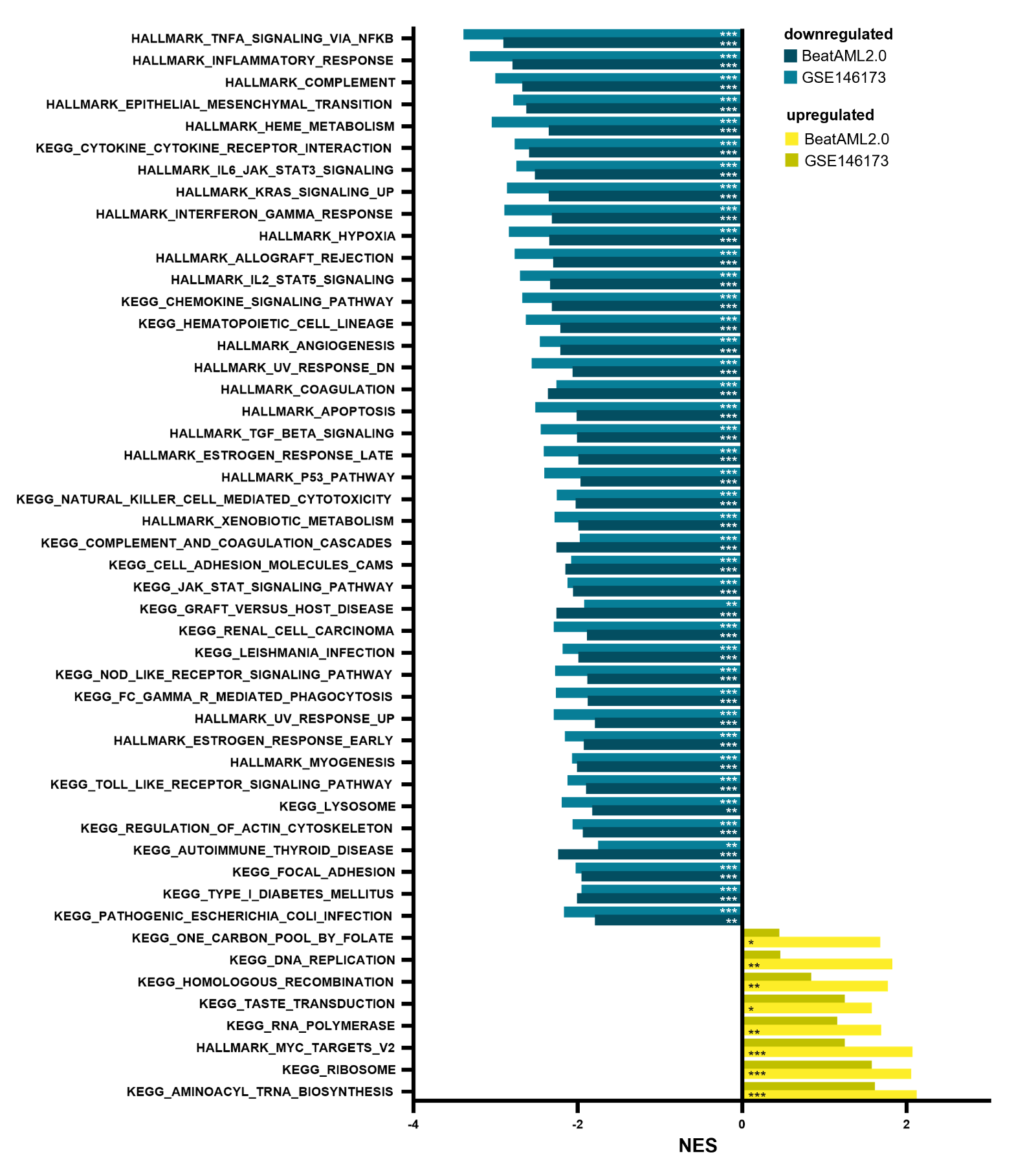


Supplementary Figure 3: Bar plot of Hallmark and KEGG pathway enrichment ranked by NES. The top 50 pathways are shown, including the top 8 upregulated and top 42 downregulated. Blue bars indicate downregulated pathways in *IL1R1*-low vs. *IL1R1*-high AML; yellow bars indicate upregulated pathways (BeatAML2.0: dark; GSE146173: light). ***p < 0.001, **p < 0.01, *p < 0.05.

**Supplementary Figure 4**: Venn diagram of inflammatory pathway genes and downregulated DEGs in *IDH1*-mut AML


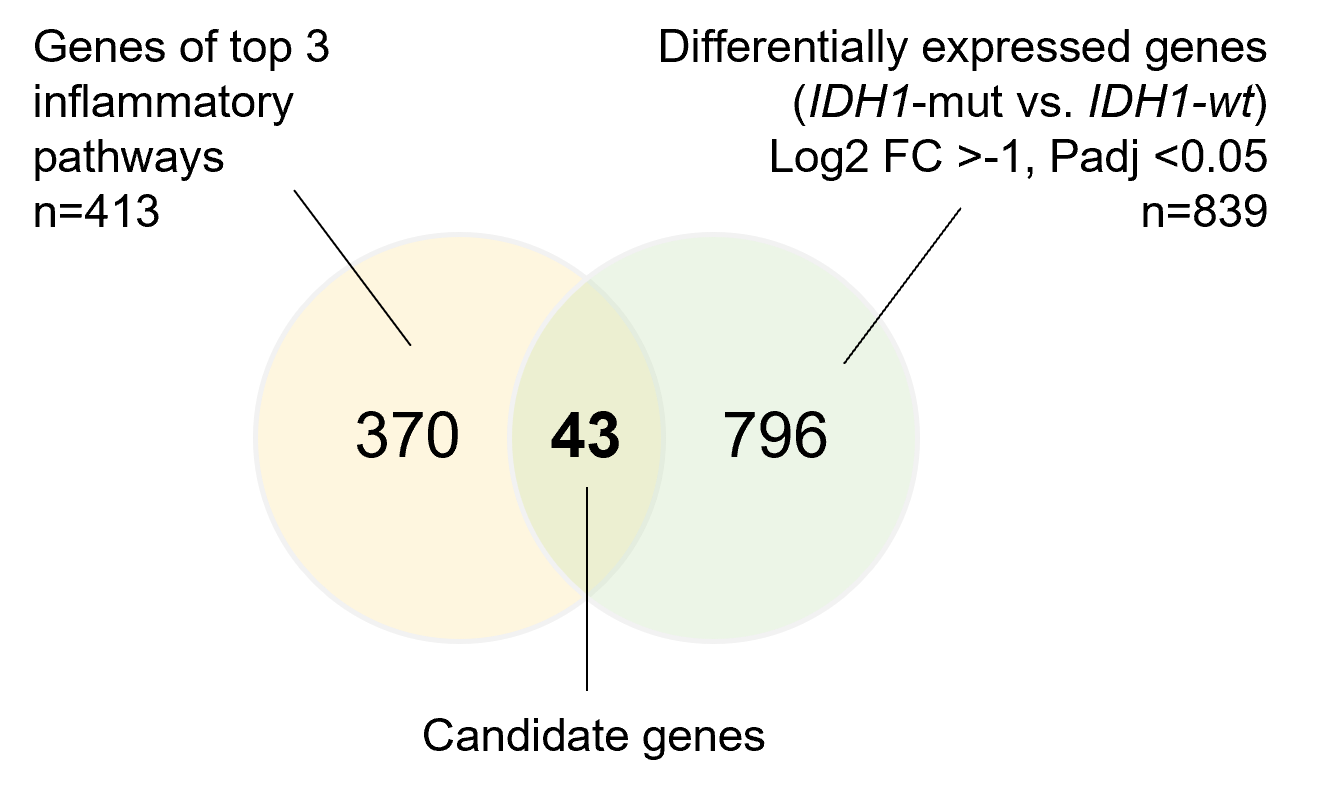


Supplementary Figure 4: Venn diagram showing the overlap between 413 genes from the top three inflammatory pathways and 839 downregulated differentially expressed genes (DEGs) identified in *IDH1*-mut versus *IDH1*-wt AML in the BeatAML2.0 cohort.

**Supplementary Figure 5**: Expression of candidate genes from selected inflammatory and immune signaling pathways across *IDH1/2*-wt, *IDH1*-mut and *IDH2*-mut AML samples


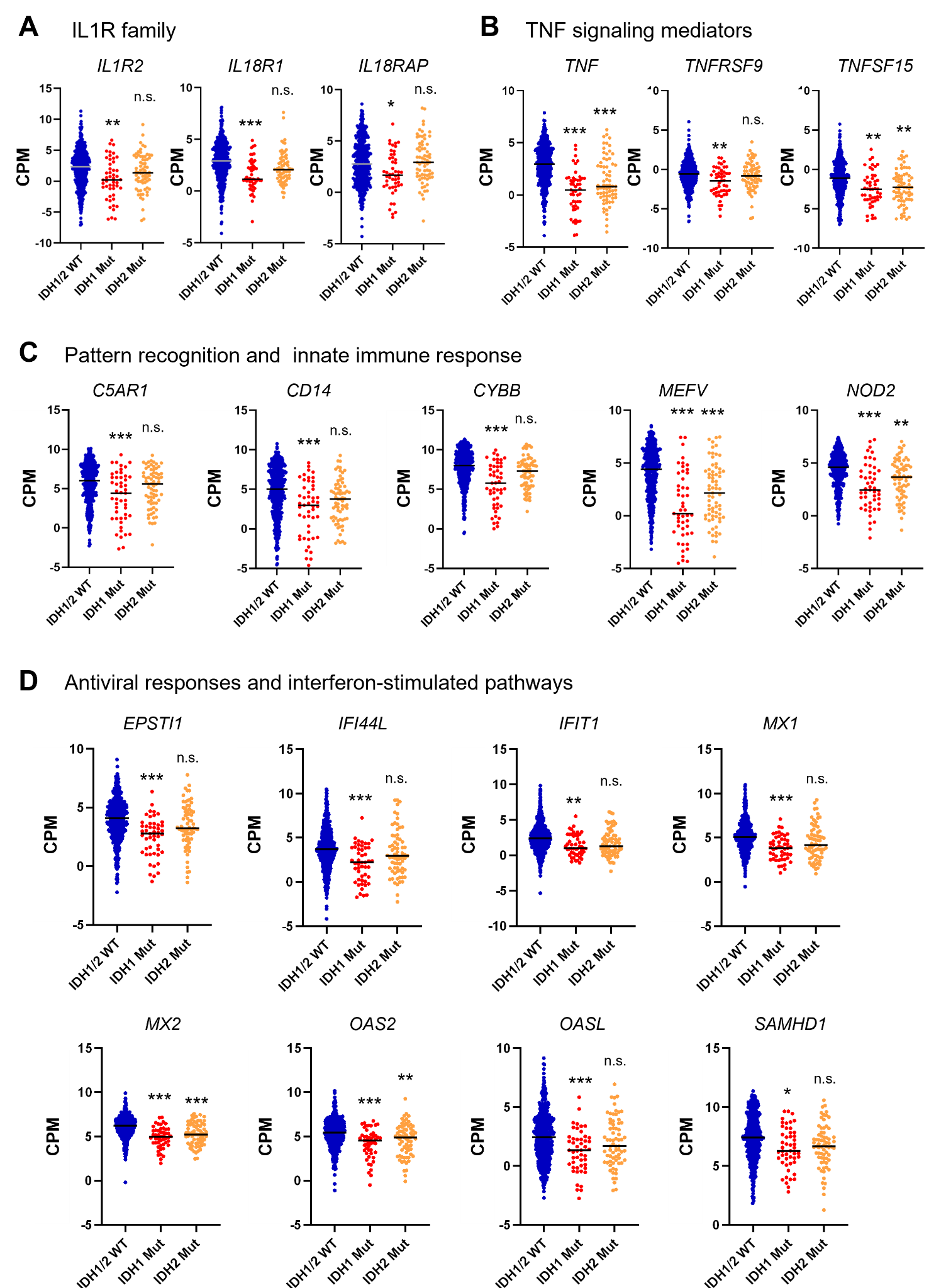


Supplementary Figure 5: Gene expression analysis from RNA-seq data (BeatAML2.0 cohort). (A) IL1R-family members including *IL1R2*, *IL18R1* and *IL18RAP*. (B) TNF signaling mediators, including *TNF*, *TNFRSF9* and *TNFSF15*. (C) Pattern recognition and innate immune response genes, including *C5AR1, CD14*, *CYBB*, *MEFV* and *NOD2*. (D) Antiviral response and interferon-stimulated genes, including *EPSTI1*, *IFI44L*, *IFIT1*, *MX1*, *MX2*, *OAS2*, *OASL* and *SAMHD1*. Each dot represents one sample (*IDH1/2*-wt, n=497; *IDH1*-mut, n=49; *IDH2*-mut, n=69). Horizontal lines indicate median CPM values. Statistical significance was assessed using the Kruskal-Wallis test followed by Dunn’s post hoc test with correction for multiple testing, with p-values indicating ***p < 0.001, **p < 0.01, *p < 0.05; n.s. = not significant.

**Supplementary Figure 6**: CpG methylation patterns of IL1R family genes in *IDH1*-mut and *IDH1*-wt AML samples


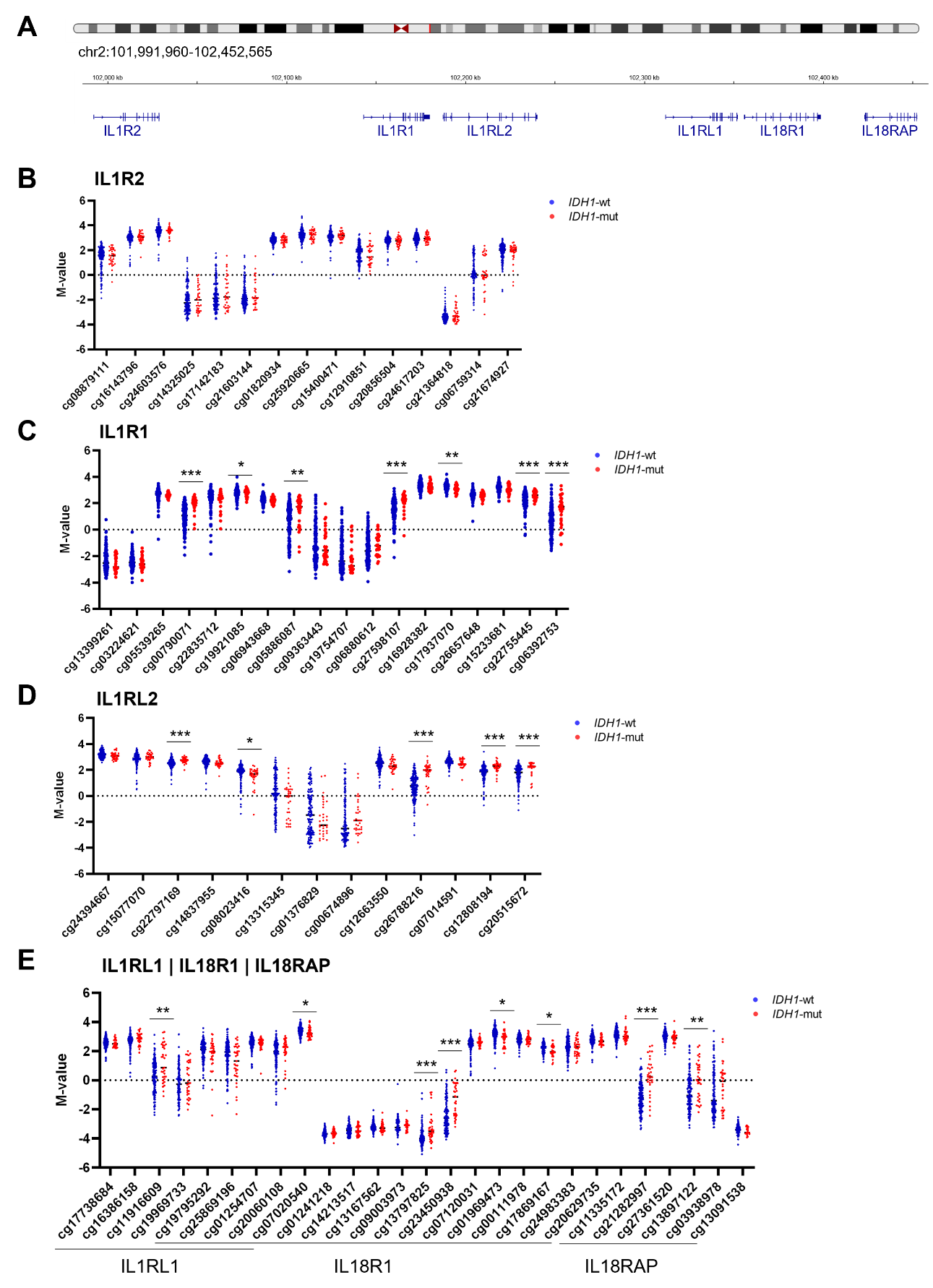


Supplementary Figure 6: (A) Schematic representation of the IL1R-family locus on chromosome 2 (chr2:101,991,960–102,452,565, hg38), including *IL1R2*, *IL1R1*, *IL1RL2*, *IL1RL1*, *IL18R1* and *IL18RAP.* (B-E) CpG methylation levels (M-values) across the IL1R-family locus obtained from Illumina 450K array profiling in (B) *IL1R2*, (C) *IL1R1*, (D) *IL1RL2* and (E) *IL1RL1*, *IL18R1* and *IL18RAP* in *IDH1*-wt (blue) and *IDH1*-mut (red) AML samples. Each dot represents an individual sample of n=146 (*IDH1*-wt) or n=33 (*IDH1*-mut).

**Supplementary Figure 7**: Treatment regimens and prognostic impact of *IDH1* genotype and *IL1R1* expression


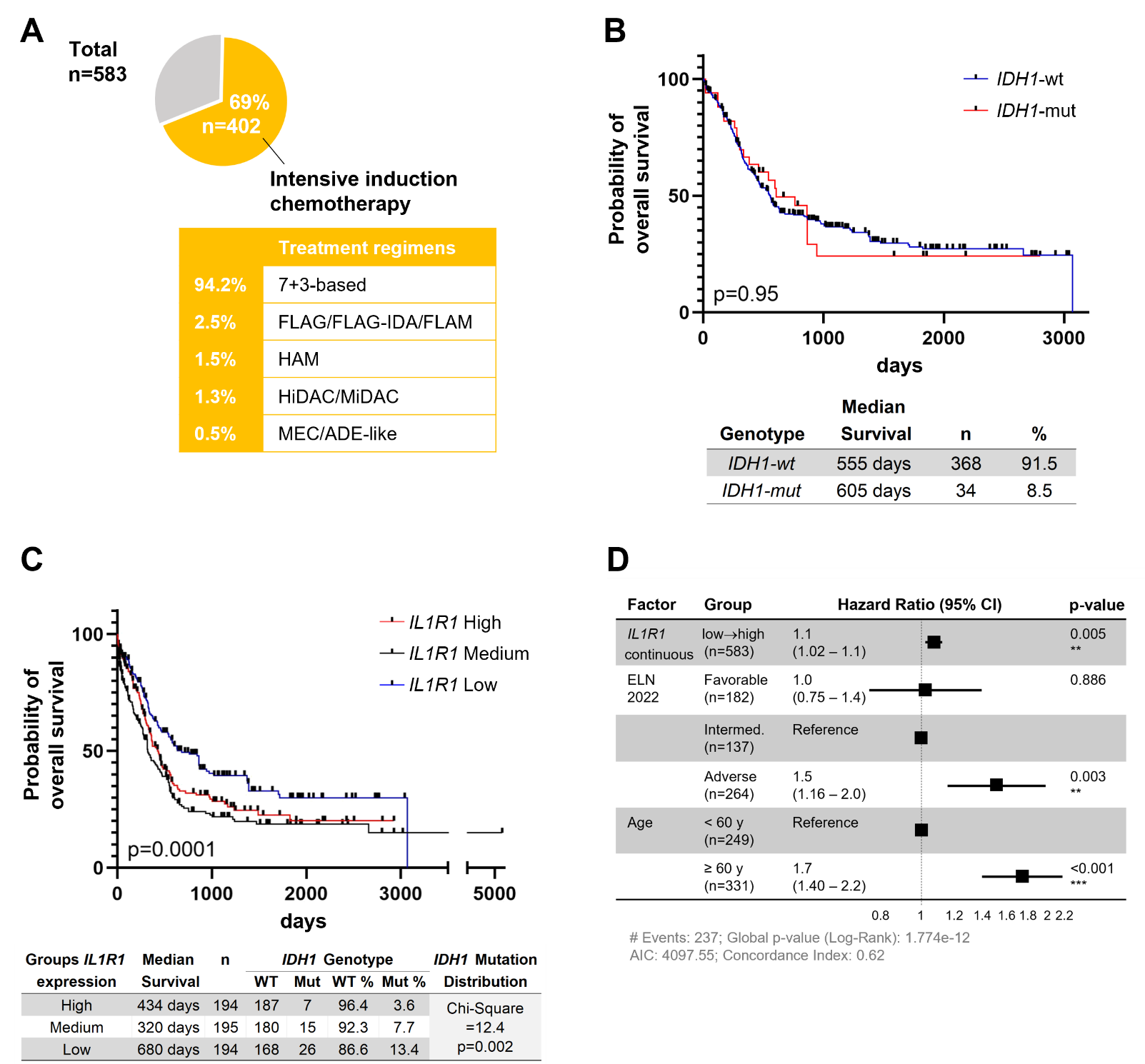


Supplementary Figure 7: (A) Distribution of treatment regimens in the BeatAML2.0 cohort (n=583). A total of 402 patients (69%) received intensive induction chemotherapy. The table summarizes the distribution of treatment regimens among intensively treated patients. (B) Kaplan-Meier analysis of overall survival comparing *IDH1*-mut and *IDH1*-wt AML. Statistical significance was assessed using the log-rank test. (C) Kaplan-Meier analysis of overall survival stratified by *IL1R1* expression tertiles. P-value was calculated using the log-rank test. The table summarizes median survival, *IDH1-*mut frequencies and mutation distribution across *IL1R1* expression groups. Differences in *IDH1-*mut frequency were assessed using the chi-square test. (D) Multivariable Cox regression analysis including *IL1R1* expression (continuous), age (≥60 vs <60 y) and ELN 2022 risk classification. Hazard ratios with 95% confidence intervals are shown.

**Supplementary Figure 8**: Transcriptomic comparison of primary *IDH1*-wt and *IDH1*-mut AML blasts


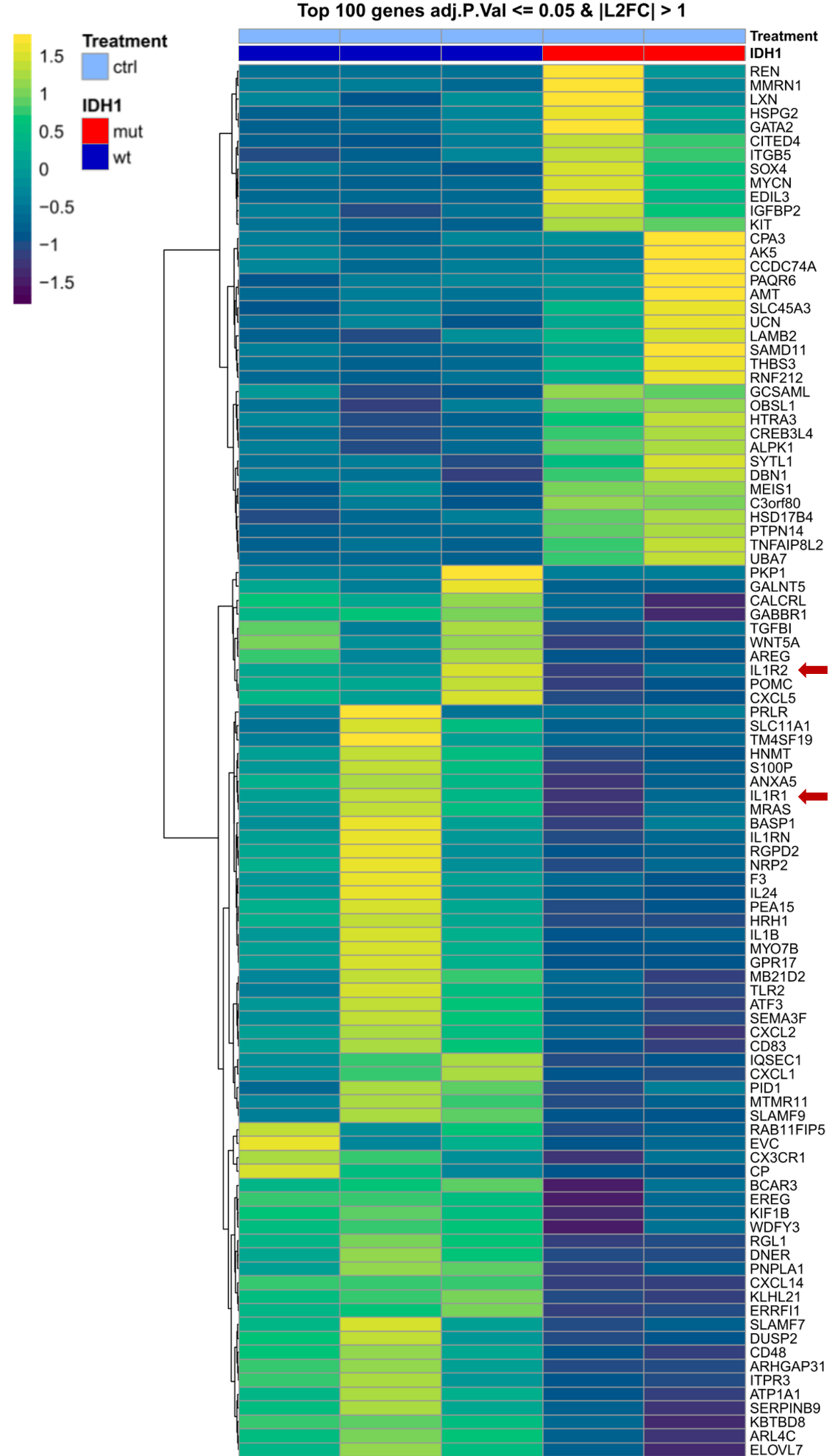


Supplementary Figure 8: Transcriptomic profiling of primary AML blasts comparing *IDH1*-wt and *IDH1*-mut samples. Shown is a heatmap of normalized expression values for the top 100 significantly differentially expressed genes (padj ≤ 0.05, log₂FC > 1). Data are based on n=3 biological replicates for *IDH1*-wt and n=2 for *IDH1*-mut.

**Supplementary Figure 9**: Inflammatory signaling of *IDH1*-wt and *IDH1-*mut primary AML blasts under IL-1β stimulation


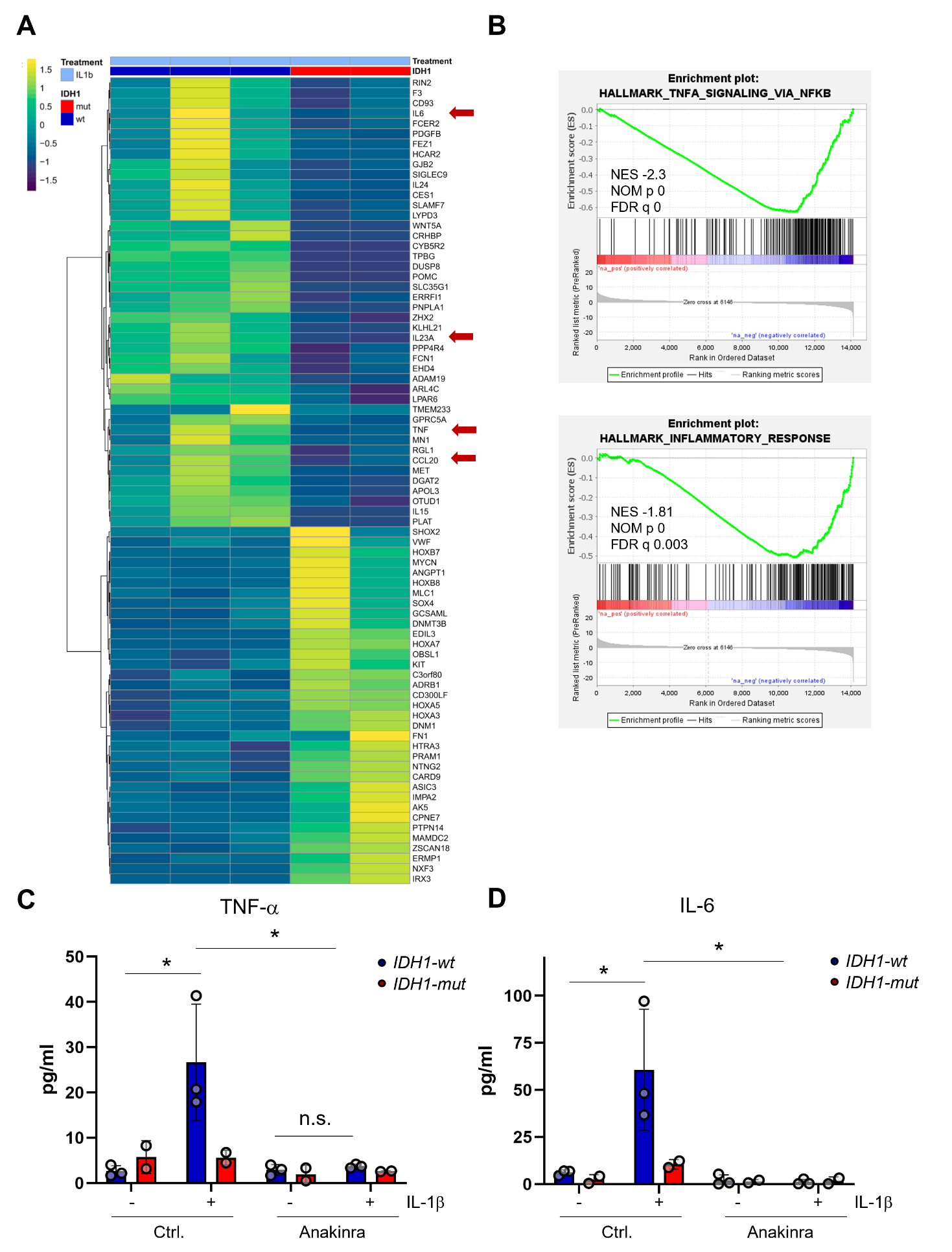


Supplementary Figure 9: (A) Differential gene expression analysis of primary AML blasts comparing *IDH1*-wt and *IDH1*-mut samples after stimulation with IL-1β (10 ng/ml for 6h). Shown are the 79 significantly regulated genes (padj ≤ 0.05, log₂FC > 1), visualized as a heatmap of normalized expression values (*IDH1*-wt n = 3; *IDH1*-mut n = 2). Red arrows indicate selected genes with known roles in TNF-α and/or inflammatory signaling. (B) Gene set enrichment plots of hallmark pathways significantly downregulated in *IDH1*-wt compared to *IDH1*-mut AML blasts in response to IL-1β stimulation (10 ng/ml for 6h), including *TNFA signaling via NFKB* (top) and *inflammatory response* (bottom). Each plot shows the NES, NOM p and FDR q-value to indicate statistical significance (FDR q < 0.05). (C, D) Quantification of secreted TNF-α (C) and IL-6 (D) in *IDH1*-wt AML blasts compared to *IDH1*-mut, under basal conditions and following IL-1β stimulation (10 ng/mL, 18 h), with or without co-treatment with the IL-1R antagonist Anakinra (10 µg/mL). Cytokine concentrations were determined by ELISA. Bars represent mean ± SD of independent biological replicates *IDH1*-wt (n = 3), *IDH1*-mut (n = 2). Statistical analysis was restricted to groups with n = 3 (*IDH1*-wt), using unpaired two-tailed t-tests; *p < 0.05, n.s. = not significant.

**Supplementary Figure 10**: GSEA of IL-1β stimulated primary AML blasts comparing *IDH1*-mut and *IDH1*-wt samples


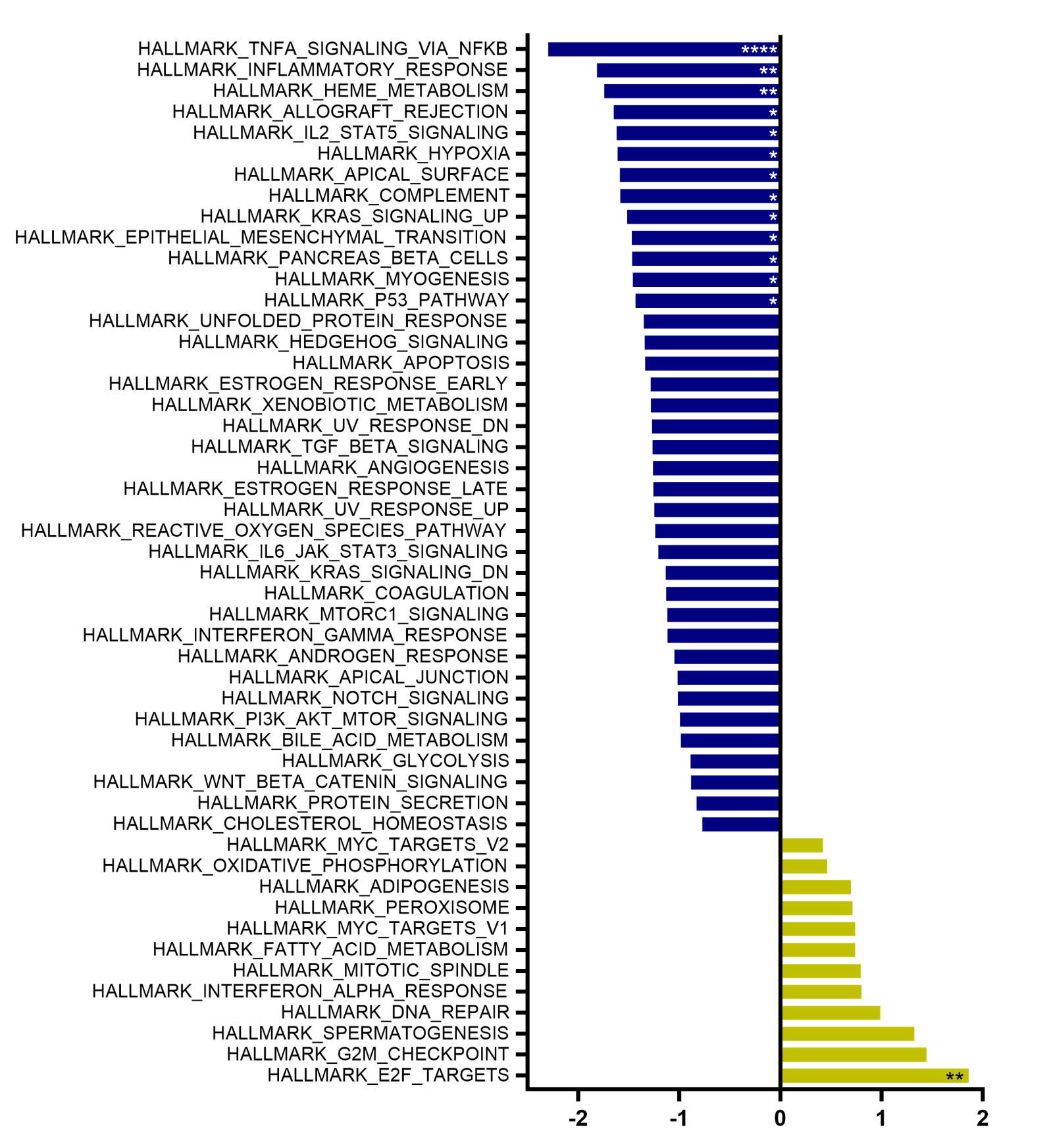


**NES**

Supplementary Figure 10: GSEA of RNA-sequencing data from primary AML blasts comparing *IDH1*-mut (n=2) and *IDH1-*wt (n=3) samples. Bar graph showing pathway enrichment analysis using Hallmark gene sets. Pathways are ranked by NES, with blue bars indicating downregulated and yellow bars indicating upregulated pathways. Only pathways with a FDR q-value below 0.05 were considered statistically significant. ****p < 0.0001, ***p < 0.001, **p < 0.01, *p < 0.05.

**Supplementary Figure 11**: *IL1R1* expression and IL-1β-induced inflammatory responses in *IDH1*-wt and *IDH1*-het KG-1a cells


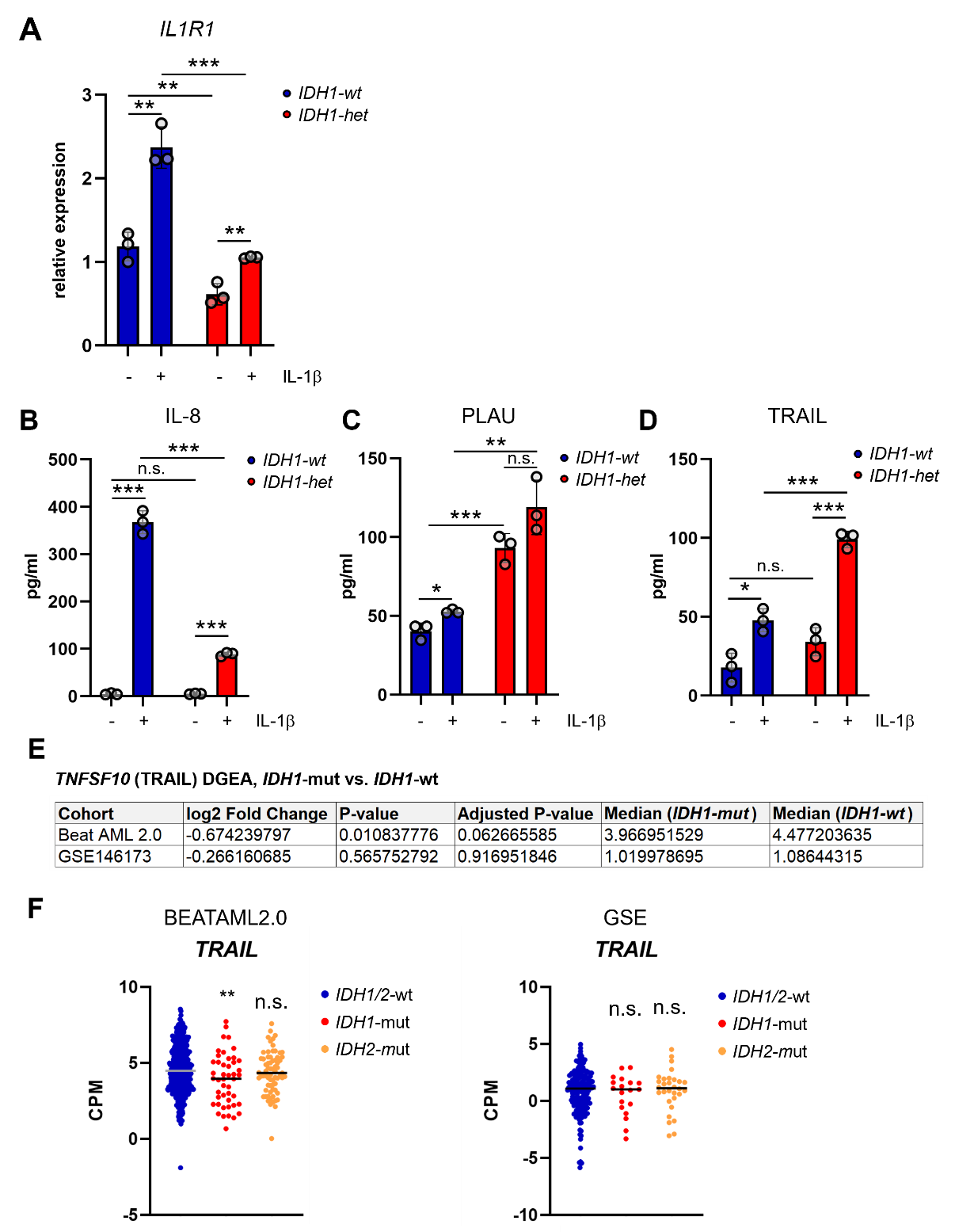


Supplementary Figure 11: (A) Relative mRNA expression of *IL1R1* in *IDH1*-wt and *IDH1*-het KG-1a cells under basal conditions and following IL-1β stimulation (10 ng/mL, 6 h), quantified by qRT-PCR. Data represent mean ± SD from three independent biological replicates. (B-D) ELISA-based quantification of IL-8 (B), PLAU (C) and TRAIL (D) secretion in *IDH1*-wt and *IDH1*-het KG-1a cell supernatants with or without IL-1β stimulation (10 ng/mL, 18 h). Bars represent mean ± SD from n=3 independent biological replicates. *p < 0.05, **p < 0.01; ***p < 0.001, n.s. = not significant.

**Supplementary Figure 12**: Cross-cohort validation of *TRAIL* expression in BeatAML2.0 and GSE146173


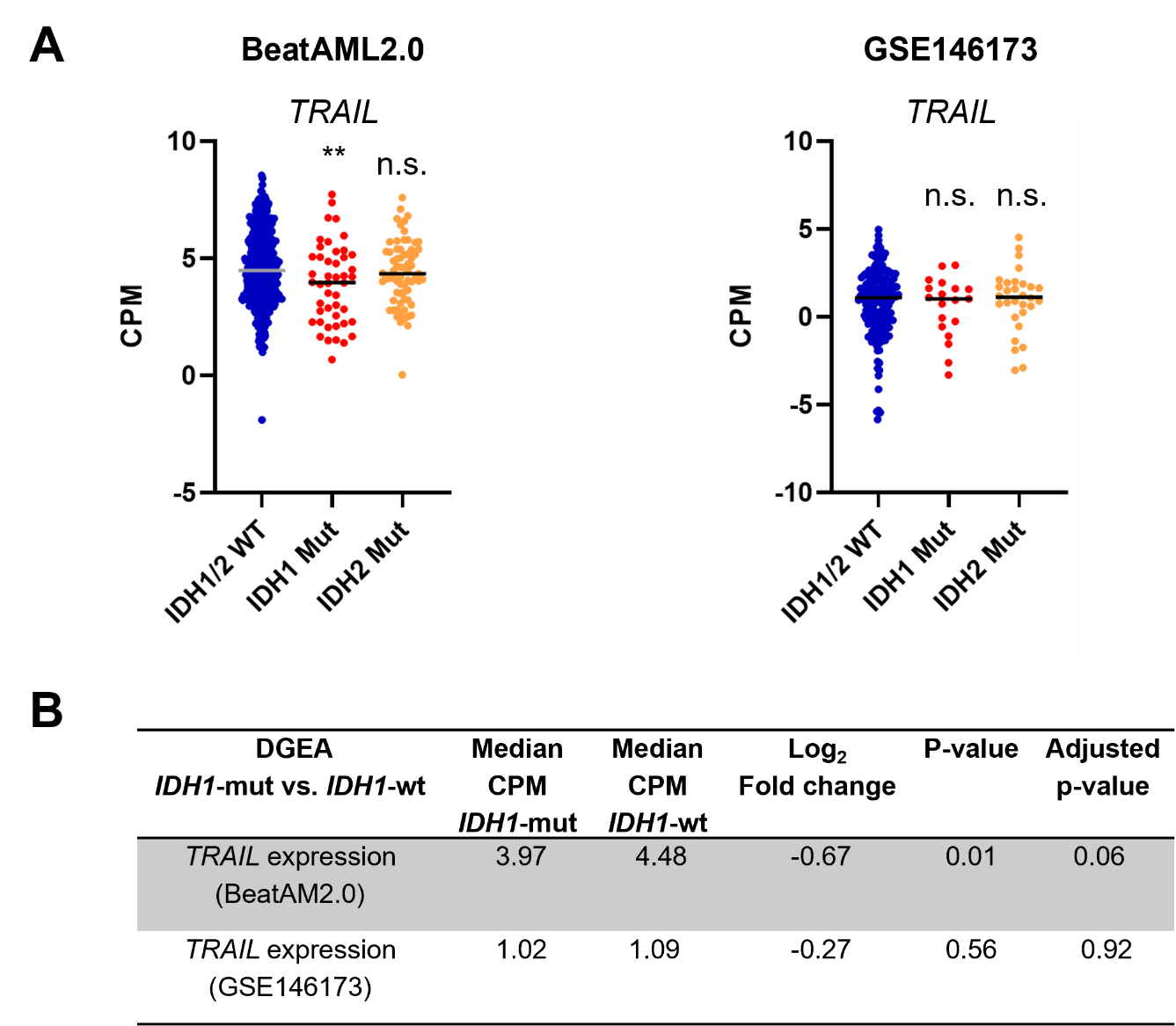


Supplementary Figure 12: (A) *TRAIL* expression across *IDH1/2*-wt, *IDH1*-mut and *IDH2-*mut AML samples in the BeatAML2.0 (left) and GSE146173 (right) cohorts. Statistical significance was assessed using two-tailed unpaired *t*-tests (**p < 0.01; n.s. = not significant). (B) Cross-cohort comparison of *TRAIL* DGEA results (*IDH1*-mut vs. *IDH1*-wt) from the BeatAML2.0 and GSE146173 datasets. Reported median CPM values, p-values and adjusted p-values correspond to those obtained from the respective DGEA.

**Supplementary Figure 13**: Assessment of apoptosis and caspase-3/7 activation in *IDH1*-wt and *IDH1*-het KG-1a cells under inflammatory conditions


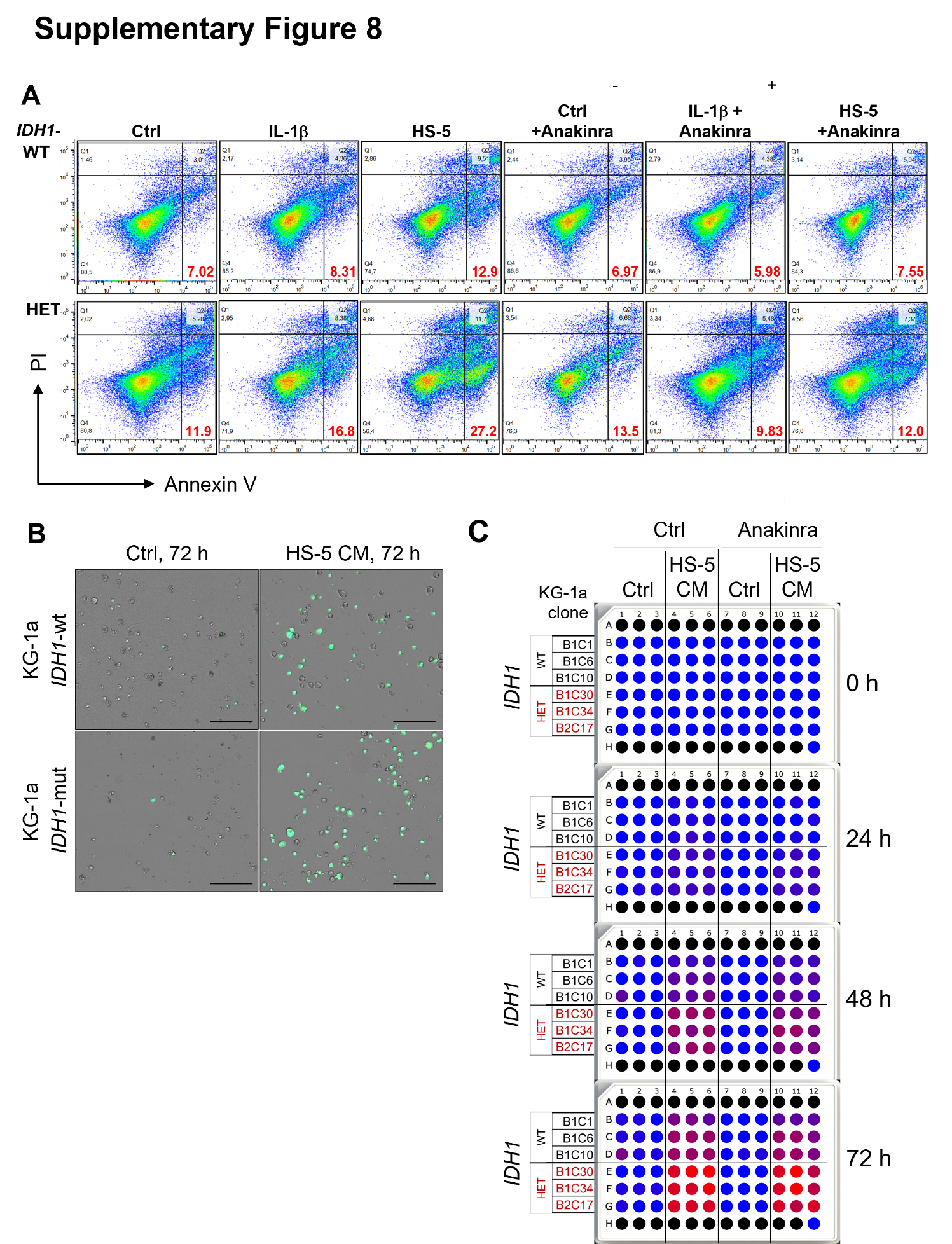


Supplementary Figure 13: (A) Representative flow cytometry plots of Annexin V/PI staining in *IDH1*-wt and *IDH1*-het KG-1a cells after 48 h of treatment with IL-1β, HS-5 conditioned media (HS-5 CM) or control conditions (Ctrl), with or without IL1R1 inhibition using 10 µg/ml Anakinra. Numbers in red indicate the percentage of Annexin V⁺/PI⁻ apoptotic cells. (B) Fluorescence microscopy images showing caspase-3/7 activation (green) in *IDH1*-wt and *IDH1*-het cells after 72 h of culture under control or HS-5 CM conditions. Scale bars represent 100 µm. (C) Heatmap from YT-SOFTWARE displaying the percentage of caspase-positive cells at different time points (0 h, 24 h, 48 h and 72 h).

**Supplementary Figure 14**: HS-5 CM-induced inflammatory protein secretion and apoptosis in *IDH1*-wt and *IDH1*-mut AML blasts


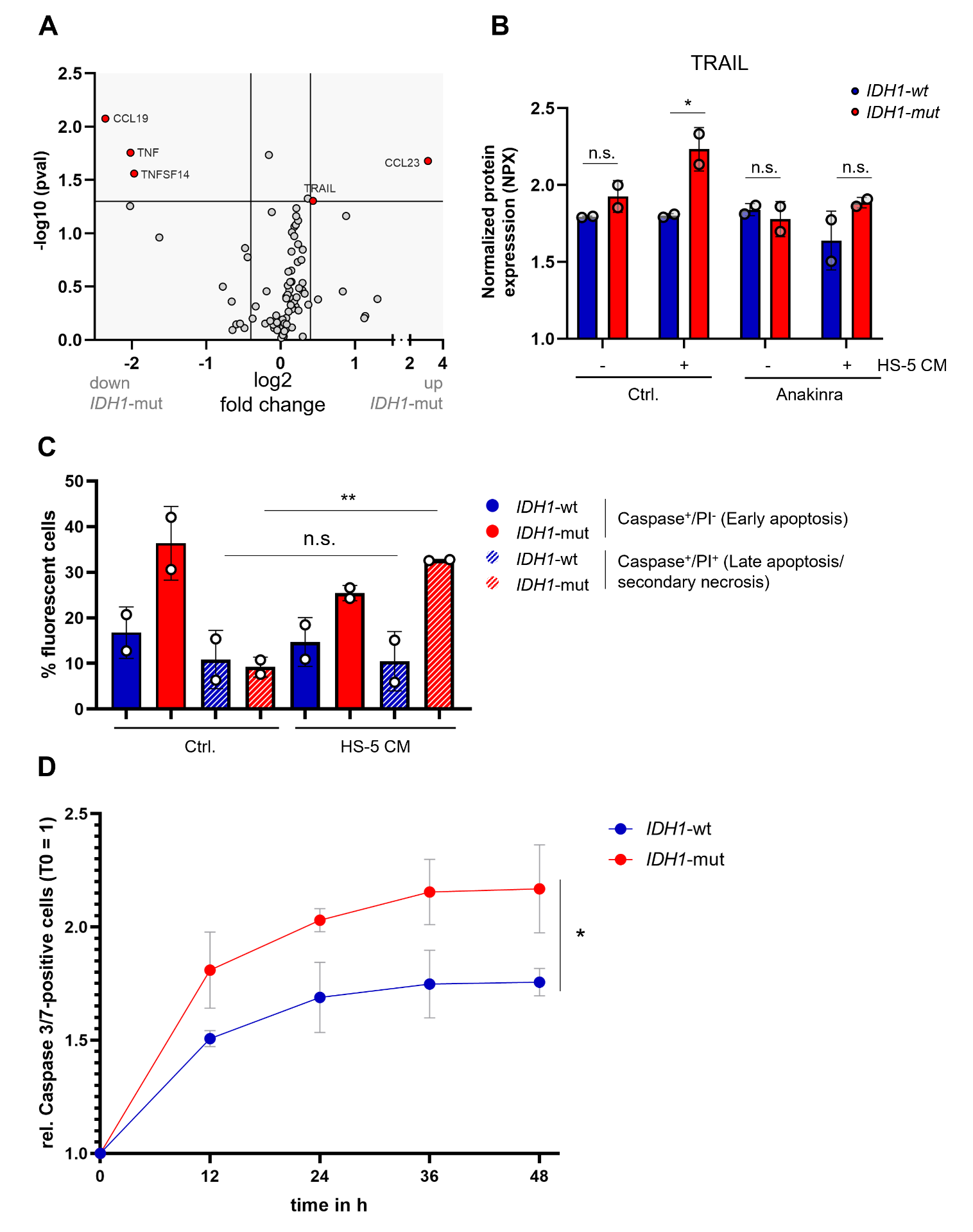


Supplementary Figure 14: (A) Volcano plot depicting significantly differentially secreted proteins in *IDH1*-mut (n=2) vs. blasts *IDH1*-wt (n=2) AML blasts following stimulation with HS-5 CM for 48 h. Protein expression was quantified using the Olink® Target 96 Inflammation panel. Horizontal and vertical lines indicate the thresholds for significance (p = 0.05) and fold change. (B) Quantification of secreted TRAIL in *IDH1*-wt and *IDH1*-mut AML blasts under basal conditions, stimulation with HS-5 CM for 48 h, with or without IL1R1 blockade using Anakinra. Bars represent mean ± SD of independent biological replicates *IDH1*-wt (n = 2), *IDH1*-mut (n = 2). Statistical analysis was performed using unpaired two-tailed t-tests; *p < 0.05, n.s. = not significant. (C) Quantification of early apoptotic (Caspase⁺/PI⁻) and late apoptotic/secondary necrotic (Caspase⁺/PI⁺) cells after 48 h of HS-5 CM stimulation in primary *IDH1*-wt and *IDH1*-mut, assessed by live-cell fluorescence imaging. Shown are percentages of early apoptotic (Caspase⁺/PI⁻) and late apoptotic or secondary necrotic (Caspase⁺/PI⁺) cells. Data represent mean ± SD of *n* = 2 independent patient samples per genotype, each measured in five technical replicates. Statistical analysis was performed using unpaired two-tailed *t*-tests. **p < 0.01; n.s. = not significant. (E) Caspase-3/7 activation was assessed over time (0 h, 12 h, 24 h, 36 h, 48 h) in primary blasts from *IDH1*-wt and *IDH1*-mut AML patients under basal conditions using fluorescence microscopy imaging. Values represent the relative percentage of caspase-3/7-positive cells, normalized to the baseline at 0 h (T0 = 1). Data are presented as mean ± SD from n = 2 biological replicates per genotype, each measured in n = 5 technical replicates. Statistical analysis was performed using two-way repeated measures ANOVA; *p < 0.05.

**Supplementary Table 1:** Primer sequences used for RT-qPCR

| **Name** | **Sequence 5’–3’** | **BP length** |
| --- | --- | --- |
| *IL1R1*_FWD1 | GTGCTTTGGTACAGGGATTCCTG | 121 |
| *IL1R1*_REV1 | CACAGTCAGAGGTAGACCCTTC | 121 |

Supplementary Table 1: Listed are the primer sequences used for RT-qPCR analysis of *IL1R1*transcripts. Primer sequences are provided in 5’–3’ direction along with the corresponding amplicon length in base pairs.

**Supplementary Table 2:** Differential gene expression analysis of *IDH1*-mut vs. *IDH1*-wt in AML samples from the BeatAML2.0 and GSE146173 cohorts

Due to its size, Supplementary Table 2 is provided as a separate file.

**Supplementary Table 3:** Pathway enrichment analysis for *IL1R1*-low vs. *IL1R1*-high AML

Due to its size, Supplementary Table 3 is provided as a separate file.

**Supplementary Table 4**: Candidate genes from the top three downregulated inflammatory pathways in *IDH1*-mut vs. *IDH1*-wt AML

Supplementary Table 4: List of 43 overlapping genes associated with the top three enriched pathways (inflammatory response, interferon gamma response and IL6/JAK/STAT3 signaling) and identified as significantly downregulated in *IDH1*-mut vs. *IDH1*-wt AML (log2FC > 1, p-adjusted < 0.05).

**Supplementary Table 5**: Differentially methylated CpG sites in the IL1R-family locus in *IDH1*-mut vs. *IDH1*-wt AML


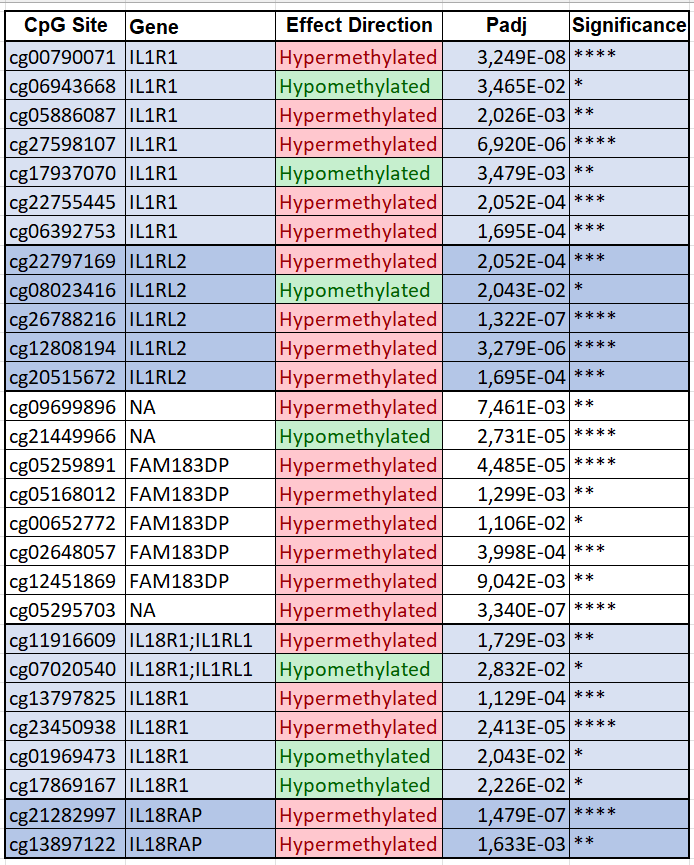


Supplementary Table 5: Differentially methylated CpG sites in the IL1R-family locus identified by Illumina 450K array profiling in *IDH1*-mut versus *IDH1*-wt AML samples. Methylation differences were evaluated using the Mann-Whitney U test, and p-values were adjusted for multiple testing with the Benjamini-Hochberg correction. Adjusted p-values (Padj) are reported, with significance levels indicated as: *Padj < 0.05, **Padj < 0.01, ***Padj < 0.001, ****Padj < 0.0001.

**Supplementary Table 6**: Genotypic and clinical characteristics of primary AML patient samples

| **ID** | **Age** | **Sex** | **BM blasts [%]** | ***IDH1* Genotype** | **Mutation** | **VAF [%]** |
| --- | --- | --- | --- | --- | --- | --- |
| Pat. 1 | 80 | m | 30 | wt | - | - |
| Pat. 2 | 49 | f | 95 | wt | - | - |
| Pat. 3 | 71 | m | 25 | wt | - | - |
| Pat. 4 | 73 | m | 90 | mut | p.R132H | 43.7 |
| Pat. 5 | 66 | m | 90 | mut | p.R132C | 47.7 |

Supplementary Table 6: Characteristics of primary AML patient samples. Shown are the sample IDs, age, patient sex, percentage of bone marrow (BM) blasts at diagnosis, *IDH1* genotype, specific mutation and percentage of variant allele frequency (VAF).

**Supplementary Table 7:** Differential gene expression analysis between *IDH1*-mut and *IDH1*-wt AML samples derived from primary AML blasts under control and IL-1β treatment conditions

Due to its size, Supplementary Table 7 is provided as a separate file.

**Supplementary References**

1. Steinhäuser S, Silva P, Lenk L, Beder T, Hartmann A, Hänzelmann S, et al. Isocitrate dehydrogenase 1 mutation drives leukemogenesis by PDGFRA activation due to insulator disruption in acute myeloid leukemia (AML). Leukemia. 2022;37(1):134–42.

2. Schmittgen TD, Livak KJ. Analyzing real-time PCR data by the comparative CT method. Nature Protocols. 2008;3(6):1101–8.

3. Wittwer CT, Vandesompele J, Shipley GL, Pfaffl MW, Nolan T, Mueller R, et al. The MIQE Guidelines: Minimum Information for Publication of Quantitative Real-Time PCR Experiments. Clinical Chemistry. 2009;55(4):611–22.

4. Hoheisel JD, Assarsson E, Lundberg M, Holmquist G, Björkesten J, Bucht Thorsen S, et al. Homogenous 96-Plex PEA Immunoassay Exhibiting High Sensitivity, Specificity, and Excellent Scalability. PLoS ONE. 2014;9(4).

5. Lundberg M, Eriksson A, Tran B, Assarsson E, Fredriksson S. Homogeneous antibody-based proximity extension assays provide sensitive and specific detection of low-abundant proteins in human blood. Nucleic Acids Research. 2011;39(15):e102–e.

6. Szklarczyk D, Kirsch R, Koutrouli M, Nastou K, Mehryary F, Hachilif R, et al. The STRING database in 2023: protein–protein association networks and functional enrichment analyses for any sequenced genome of interest. Nucleic Acids Research. 2023;51(D1):D638–D46.

7. Bottomly D, Long N, Schultz AR, Kurtz SE, Tognon CE, Johnson K, et al. Integrative analysis of drug response and clinical outcome in acute myeloid leukemia. Cancer Cell. 2022;40(8):850–64.e9.

8. Herold T, Jurinovic V, Batcha AMN, Bamopoulos SA, Rothenberg-Thurley M, Ksienzyk B, et al. A 29-gene and cytogenetic score for the prediction of resistance to induction treatment in acute myeloid leukemia. Haematologica. 2018;103(3):456–65.

9. Kaplan EL, Meier P. Nonparametric Estimation from Incomplete Observations. Journal of the American Statistical Association. 1958;53(282).
